# Effects of a cardiopulmonary rehabilitation programme on submaximal exercise in Tunisian patients with long-COVID19: A randomized clinical trial

**DOI:** 10.5114/biolsport.2024.139072

**Published:** 2024-04-25

**Authors:** Rania Kaddoussi, Hadhemi Rejeb, Amine Kalai, Eya Zaara, Naceur Rouetbi, Zohra Ben Salah Frih, Piotr Zmijewski, Helmi Ben Saad

**Affiliations:** 1 Department of Pneumology, Fattouma Bourguiba Hospital, Monastir, Tunisia; 2 Ibn Nafiss department of Pneumology, Abdelrahman Mami hospital, Ariana, Tunisia; 3 Department of Physical Medicine and Rehabilitation, Fattouma Bourguiba Hospital, Monastir, Tunisia; 4 Jozef Pilsudski University of Physical Education in Warsaw, Warsaw, Poland; 5 Heart Failure (LR12SP09) Research Laboratory, Farhat HACHED Hospital, Sousse, Tunisia; 6 Laboratory of Physiology. Faculty of Medicine of Sousse. University of Sousse, Sousse Tunisia; 7 Department of Physiology and Functional Exploration. Farhat HACHED Hospital, Sousse, Tunisia

**Keywords:** Ambulatory cardiopulmonary rehabilitation, Long-term COVID-19 effects, MCID, Persistent COVID-19 symptoms, Post-acute COVID-19, Randomized clinical trial, Submaximal exercise capacity, Therapeutic education

## Abstract

There is a lack of randomized clinical trials (RCTs) exploring the outcomes of cardiopulmonary rehabilitation programmes (CPRPs) on submaximal aerobic capacity of long COVID-19 patients (LC19Ps). This RCT aimed to evaluate the effect of an ambulatory CPRP on the 6-min walk test (6MWT) data (main outcome: 6-min walk distance (6MWD)) of LC19Ps. Conducted as a single-blinded RCT, the study included Tunisian LC19Ps with persistent dyspnoea (i.e. modified medical research council (mMRC) level ≥2) at least three months postdiagnosis. LC19Ps were randomly assigned to the intervention (IG, n = 20) or control (CG, n = 10) groups. Pre- and post-CPRP evaluations included dyspnoea assessments (Borg and mMRC scales), anthropometric data, spirometry, and 6MWT. The CPRP (i.e. 18 sessions over six weeks) encompassed warm-up, aerobic training, resistance training, respiratory exercises, and therapeutic education. The CPRP significantly improved i) dyspnoea, i.e. IG exhibited larger reductions compared to the CG in Borg (-3.5 ± 2.0 vs. -1.3 ± 1.5) and mMRC (-1.5 ± 0.8 vs. -0.1 ± 0.3) scales, and ii) 6MWD, i.e. IG demonstrated larger improvements compared to the CG in 6MWD (m, %) (168 ± 99 vs. 5 ± 45 m, 28 ± 8 vs. 1 ± 8%, respectively), and resting heart rate (bpm, % maximal predicted heart rate) (-9 ± 9 vs. 1 ± 7 bpm; -5 ± 6 vs. 0 ± 4%, respectively), with small effect sizes. In the IG, the 1.5-point decrease in mMRC and the 168 m increase in 6MWD exceeded the recommended minimal clinical important differences of 1 point and 30 m, respectively. CPRP appears to be effective in enhancing the submaximal exercise capacity of LC19Ps, particularly in improving 6MWD, dyspnoea, and resting heart rate. RCT registration: www.pactr.org; PACTR202303849880222.

## LIST OF ABBREVIATIONS

ANOVA: analysis of variance

ATS: American Thoracic Society

BMI: body mass index

CG: control group

COVID-19: coronavirus disease 2019

CPRPs: cardiopulmonary rehabilitation programmes

DBP: diastolic blood pressure

_End_: after the walk

ERS: European Respiratory Society

FEV_1_: forced expiratory volume in one second

FVC: Forced vital capacity

HR: heart rate

IG: intervention group

LC19: long COVID-19

LC19Ps: LC19 patients

LLN: lower limit of normal

MCID: minimal clinically important difference MD: mean difference

mMRC: modified medical research council

PMHR: predicted maximal HR

RCTs: randomized clinical trials

_Rest_: before the walk

RT-PCR: real-time reverse-transcription polymerase chain reaction

SARS-CoV-2: severe acute respiratory syndrome coronavirus-2 infection

SBP: systolic blood pressure

SpO_2_: oxy-haemoglobin saturation

SD: standard deviation

SR: systematic review

6MWD: 6-min walk distance

6MWT: 6-min walk test

6MWW: 6-min walk work

ΔExercise: delta exercise change (= 6MWT_End_ value minus 6MWT_Rest_ value)

## INTRODUCTION

The coronavirus disease 2019 (COVID-19) pandemic has had a profound global impact, causing widespread economic disruption and social upheaval [1]. As of February 19, 2024, there have been a staggering 70,3482,641 reported COVID-19 infections and 6,984,597 related deaths (0.9928%) worldwide (https://www.worldometers.info/coronavirus/). COVID-19, caused by severe acute respiratory syndrome coronavirus-2 infection (SARS-CoV-2), affects the respiratory system and various extra pulmonary organs [2]. Manifestations range from asymptomatic to critical illness [3]. Pneumonia resulting from COVID-19 infection can lead to permanent lung parenchyma structural damage, even with appropriate medical treatment [4]. Consequently, most COVID-19 patients continue to experience sequelae and medical complications lasting weeks to months after initial recovery [5]. In the post-acute phase of COVID-19, 40% to 90% of patients continue to manifest symptoms for months [5]. The term long COVID-19 (LC19) (also called “longterm COVID-19 effects”, “post-acute COVID-19”, and “persistent COVID-19 symptoms”), characterized by the continuation or development of new symptoms three months after the initial infection with SARS-CoV-2, refers to symptoms lasting at least two months with no other explanation [5, 6]. LC19 presents with diverse clinical manifestations affecting various systems, including respiratory, cardiovascular, muscular, nutritional status, and sleep [5, 7, 8]. First, studies on LC19 patients (LC19Ps) reported persistent reductions in forced expiratory volume in one second (FEV_1_) and forced vital capacity (FVC), as well as exertional dyspnoea (modified medical research council (mMRC)) [9, 10]. Second, a 2023 systematic review (SR) and meta-analysis revealed significantly higher odds ratios for cardiovascular outcomes in LC19Ps compared to controls, including electrophysiological abnormalities, coronary vessel diseases, thromboembolic disorders, and diseases of cardiac tissue [11]. Third, musculoskeletal complaints were reported in 39% of LC19Ps [7]. Fourth, a study reported that 13.3% of COVID symptom study app users experienced at least one persistent symptom beyond four weeks of infection, with half believed to be cardiac in origin [12]. Finally, a reduced 6-min walk distance (6MWD) was reported by some authors [9], and it appears that approximately 33% [13] and 23% [14] of LC19Ps have a decreased peak oxygen consumption three and 12 months after hospital discharge, respectively. Given the significant compromises in daily functioning experienced by survivors of moderate to severe COVID-19 [13], cardiopulmonary rehabilitation programmes (CPRPs) have emerged as a crucial intervention for improving outcomes in LC19Ps and preventing further long-term damage [15–19].

CPRPs are pivotal in managing chronic cardiorespiratory conditions, with well-established benefits [20]. A 2022 American consensus specifically addressed post-acute persistent breathing discomfort and respiratory sequelae in LC19Ps, recommending CPRPs for those with dyspnoea, breathing abnormalities, fatigue, peripheral and respiratory muscle weakness, and reduced endurance [21]. The aim is to promote functional improvement and facilitate a return to daily activities [21]. CPRPs have demonstrated safety and effectiveness, even in severe LC19Ps [19]. Assessing submaximal exercise capacity through the 6-min walk test (6MWT) is recommended for patients with cardiorespiratory conditions [22]. The 6MWT is widely used in CPRPs, providing valuable insights into their impact on cardiorespiratory fitness [23]. A 2022 SR [24] concluded that CPRPs had inconsistent results in pulmonary function of LC19Ps. Nevertheless, the SR detected improvements in dyspnoea, muscle strength, 6MWD, and quality of life [24]. However, since this SR [24] included a limited number of randomized clinical trials (RCTs) (n = 5), with two having a low risk of bias and three in the “some concerns” category, additional RCTs are needed to confirm these preliminary findings. This was corroborated by a 2023 SR [25] assessing the impact of CPRPs on 6MWT outcomes in LC19Ps, which included six RCTs demonstrating “low” or “moderate” risk of bias [26–31]. The SR concluded that CPRPs show promise in improving submaximal exercise performance among LC19Ps, and further research is needed to refine these programmes [25]. As of December 30, 2023, 26 RCTs related to the effects of CPRPs on LC19Ps have been published in *PubMed* [i.e. research using the two keywords (pulmonary rehabilitation) AND (COVID-19)] (see all references in the Appendix), but only 11 have exclusively reported 6MWT and/or pulmonary function data, among others [26–36]. Among these 11 RCTs, two [27, 28] opted for CPRPs exclusively via telemedicine, three [26, 30, 32] opted for both hospital and telemedicine CPRPs, and six [29, 31, 33–36] opted for exclusively hospital CPRPs. In North Africa, it appears that only one Egyptian RCT has evaluated the effects of a CPRP on LC19Ps [29]. The authors determined whether the addition of manual diaphragm release to an inspiratory muscle-training programme is more effective than inspiratory muscle training alone in reducing blood pressure, dyspnoea, fatigue, and aerobic performance capacity in male LC19Ps [29]. In Tunisia, only one pilot observational study has evaluated the effects of a CPRP on submaximal exercise capacity [37] social disadvantage and physical activity data [38] of male LC19Ps.

The main objective of the present RCT conducted in Tunisia was to evaluate the impacts of an ambulatory CPRP on submaximal aerobic capacity, evaluated via 6MWT data (the main outcome is the 6MWD). The null hypothesize is that the two groups (i.e. intervention (IG) and control (CG) groups) will have comparable 6MWDs. The CPRP will be considered ‘efficient’ if the change in 6MWD exceeds the recommended minimal clinically important difference (MCID) of 30 m [39]. The secondary aim was to evaluate the effects of the CPRP on perceived dyspnoea and spirometric data. A decrease of more than one point for mMRC dyspnoea will signify a perceived clinical improvement [40, 41]. The last aim was to perform a narrative review including exclusively RCTs aiming to determine the effectiveness of CPRPs on the submaximal exercise capacity of LC19Ps, specifically assessed through the 6MWT data.

## MATERIALS AND METHODS

**FIG. 1:**
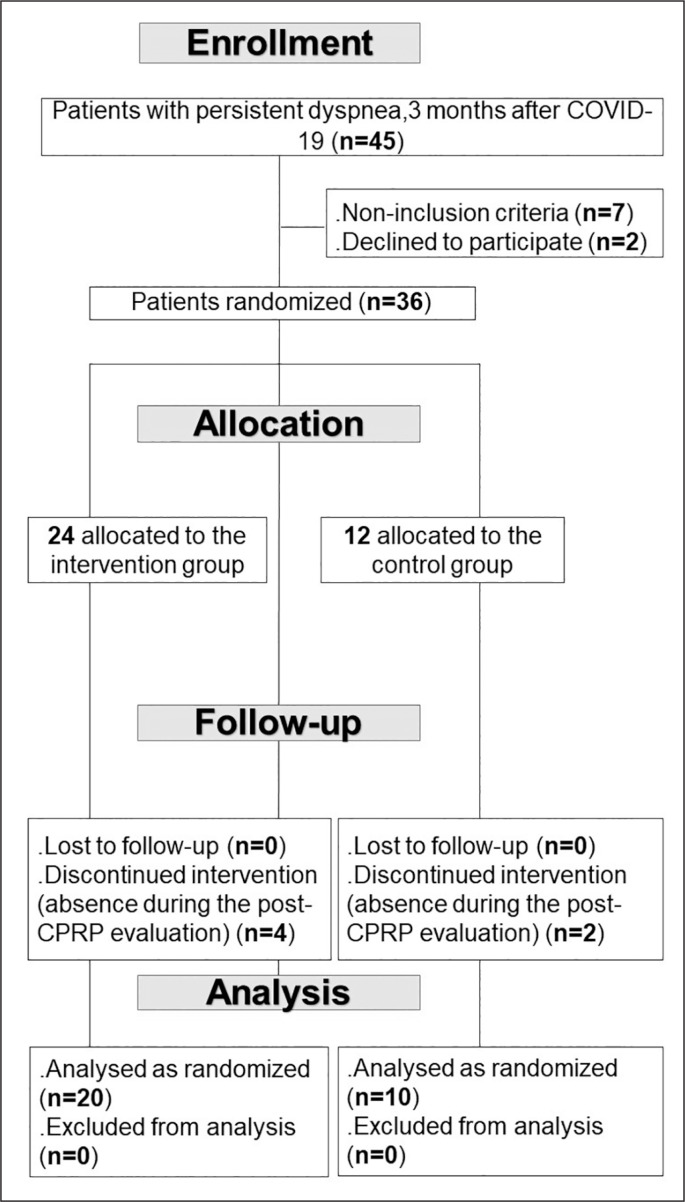
Study protocol. COVID-19: coronavirus disease 2019. CPRP: cardiopulmonary rehabilitation programme

This study is one component of a broader project comprising two distinct parts. The first part constitutes the aim of this RCT. The second part will involve evaluating the effects of CPRP on social disadvantage, encompassing psychological data and health-related quality of life. Figure 1 details the flowchart of the current project.

### Study design

This was a single-blinded RCT carried out by two Tunisian teams from the departments of pulmonology and physical medicine and rehabilitation (Fattouma Bourguiba hospital, Monastir, Tunisia). This study was approved by the medical and research ethics committee of the faculty of medicine of Sousse (Approval number CEFMS 162/2023). The study was registered at the Pan African Clinical Trial Registry (www.pactr.org; PACTR202303849880222). A written informed consent form was issued and signed by all patients before their inclusion in the study. Clear and appropriate information was communicated to each patient in French or in Arabic languages, including the description and the progress of the various examinations.

**FIG. 2:**
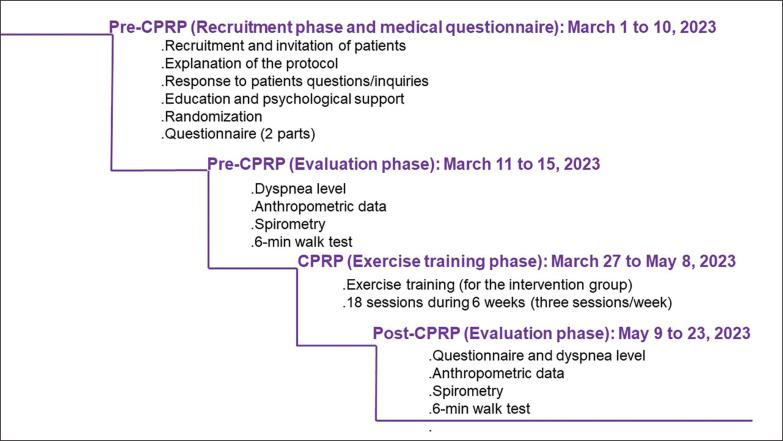
Description of the 4 phases of the cardiopulmonary rehabilitation programme (CPRP).

This study was performed during a period of 84 days (i.e. from April 1 to June 23, 2023 (Figure 2]). A ten-day period (i.e. from April 1 to 10) was reserved for the recruitment of LC19Ps (i.e. invitation to participate, explanation of the protocol and answering patients’ questions). During a two-week period (i.e. April 11 to April 25, 2023), the pre-CPRP evaluations’ tests were performed. A six-week period (i.e. April 27 to June 8, 2023) was reserved for the practice of the CPRP. During a two-week period (i.e. June 9 to 23, 2023), the post-CPRP evaluations’ tests were performed.

### Study population

The source population for this study comprised LC19Ps residing in Monastir (Tunisia), who sought care at the outpatient department of pulmonology in the mentioned hospital. Inclusion criteria encompassed patients with a confirmed diagnosis of COVID-19, aged over 18 years, experiencing persistent dyspnoea three months after the COVID-19 diagnosis, with dyspnoea scoring two or more on the mMRC scale [42]. LC19Ps with pre-existing chronic lung conditions such as asthma, chronic obstructive pulmonary disease, or lung cancer, those with moderate to advanced heart failure, patients with conditions affecting walking or limiting mobility (e.g. orthopaedic, rheumatologic, or muscular diseases), active cigarette or narghile smokers, and those having contraindications to the 6MWT [22, 39, 43] (e.g. signs of unstable angina or myocardial infarction within the previous month, resting (_Rest_) heart rate (HR_Rest_) ≥ 120 bpm, systolic blood pressure (SBP_Rest_) ≥ 180 mmHg, diastolic blood pressure (DBP_Rest_) ≥ 100 mmHg)) or to spirometry [44] were not included in the study. Files of patients who missed any session of the CPRP or did not attend the final evaluation were excluded from the final statistical analysis.

### Random assignment and blinding

Patients were randomly assigned to either undergo the CPRP (i.e. IG) or not (i.e. CG). The random allocation sequence was generated using free software (http://www.randomized.org/; last visit: February 24, 2024). The principal investigator (*RK* in the authors’ list) executed the randomization sequence to determine two groups identified as IG and CG. The evaluator who enrolled and assessed patients (*EZ* in the authors’ list) had no access to the randomization sequence.

The investigators (*EZ* and *HR* in the authors’ list) and patients in the study were blinded throughout the entire procedure. The investigators were uninformed about the study aims and the randomized distribution of patients to study groups, and they did not have access to the randomization sequence. Meanwhile, although blinding for patients could not be achieved, patients were unaware of the other treatment modalities. They did not know if they belonged to the IG or CG.

### Sample size estimation

The sample size was estimated using the predictive formula [45]:

– N = [(r+1) (*Z*_α/2 +_ *Z*_1−β_)^2^ δ^2^]/(r d^2^), where
– *“N”* is equal to *n*_1_ *+ n*_2_ (i.e.; sample sizes for the CG and IG);
– *“Z*_α/2_” is the normal deviate at a 5% level of significance (= 1.96);
– “*Z*_1−β_” is the normal deviate at 90% statistical power with 10% type II error (= 1.28);
– “*r”* (equal to *n*_1_*/n*_2_) is the ratio of the sample size required for the two groups (here, r = 0.5 gives a sample size distribution of 0.5:1 for the CG and IG);
– “δ” and “*d*” are the pooled standard deviation (SD) and the difference of the main outcome (i.e. change in the 6MWD after CPRP).

Given the pioneering nature of this study at the time it was conducted, values of “δ” and “*d”* were obtained from a previous RCT [46] evaluating the feasibility and effectiveness of a CPRP through telerehabilitation tools in COVID-19 patients with mild to moderate symptomatology in the acute stage (18 in the IG and 18 in the CG). The 6MWD mean ± SD changes were 80 ± 126 and 0.05 ± 26 m, in the IG and CG, respectively [46]. Insertion of the aforementioned data into the formula resulted in a total sample of 28 patients (19 in the IG and 9 in the CG). Assuming a 20% absence during the CPRP or the post-CPRP evaluation session, the revised sample size was calculated to be 35 patients [35 = 28/ (1–0.20)].

### COVID-2019 diagnosis and extent evaluation

The diagnosis of COVID-19 was confirmed by the presence of a real-time reverse-transcription polymerase chain reaction (RTPCR). Before commencing the CPRP, a chest scan was performed for all patients to determine the extent of parenchymal lung injury. The evaluation categorized patients into five groups based on the extent of lung involvement: absent or minimal (< 10%), moderate (10–25%), extensive (25–50%), severe (50–75%), and critical (> 75%) [47].

### Phases of the study and applied protocol

Figure 2 summarizes the four phases of the study.

### First phase (10 days): Recruitment phase and medical questionnaire

This initial phase focused on recruiting LC19Ps during their consultations in the outpatient department of pulmonology. During the recruitment process (i.e. meeting), the investigators (RK and AK in the authors’ list) explained the protocol’s content, progression, and tasks to be carried out during different phases to the patients. They addressed patient questions regarding the project’s aims, methods of data collection and usage, as well as participation modalities. The meeting also served to assess the patient’s general aptitude, offer advice on managing comorbidities such as diabetes mellitus and arterial hypertension, and introduce psychological support (e.g. handling emotional distress, post-traumatic stress disorder, and strategies for coping with COVID-19) [48], along with nutritional counselling [49]. Following this, patients signed the consent form, and randomization was conducted. At the phase’s conclusion, an interviewer (EZ in the authors’ list), unaware of the patient’s group allocation, completed a questionnaire for each patient. The questionnaire, with an average duration of approximately 30–40 minutes, was administered by the same interviewer before and after CPRP. In Tunisian dialect, the questionnaire comprised two parts. The first part, a general questionnaire, covered demographic (e.g. age, sex, socioeconomic level, smoking status), clinical (e.g. lifestyle habits, medical history), and COVID-19-related information (e.g. date of RT-PCR, hospitalization details, length of hospitalization, symptom duration, treatment such as corticosteroid or oxygen therapies, imaging, and extent of lesions). Low socioeconomic level was defined as unskilled worker, jobless, and high socioeconomic level as skilled worker, farmer and manager. History of cigarette smoking was evaluated in pack-years, and patients were classified into non-smoker, passive smoker, and ex-smoker. Narghile smoking was also assessed. The second part of the questionnaire was reserved to explore patients’ health-related quality of life and psychological data. The data of this part will be examined in the second phase of the project.

### Second phase (2 weeks): Pre-CPRP evaluations

During this phase, the interviewers (HR and EZ in the authors’ list) received the LC19Ps in groups of four or five per day, and the following four evaluations/tests were performed on the same day in the morning, and in the following order: dyspnoea, anthropometric data, spirometry test, and 6MWT.

Dyspnoea was assessed before and after CPRP using two scales: mMRC [42] and Borg [50] scales. Questions were presented in Arabic language. The mMRC scale, a self-rating scale, measures the disability caused by breathlessness in daily activities, ranging from level 0 to level 4 [42] (details in the Appendix). The Borg scale was used to rate dyspnoea from 6 (i.e. no exertion at all) to 20 (i.e. maximal exertion) and to monitor patients’ post-CPRP progress [50]. For the pre-CPRP evaluation phase, patients were asked to evaluate their mMRC dyspnoea level before and during the COVID-19, and whether their dyspnoea was subjectively worsened.

Anthropometric data (e.g. age (year), height (cm), weight (kg), and body mass index (BMI, kg/m^2^) were determined. Corpulence status (i.e. underweight (BMI < 18.5 kg/m^2^), normal weight (BMI: 18.5–24.9 kg/m^2^), overweight (BMI: 25.0–29.9 kg/m^2^), and obesity (BMI ≥ 30.0 kg/m^2^)) was noted [51].

The spirometry test was conducted by an experienced technician using a portable spirometer (MIR, Spirodoc, Italy) following international guidelines [44]. The collected spirometric data (i.e. FVC (L), FEV_1_ (L), FEV_1_/FVC ratio (absolute value)) were expressed as absolute values, percentages of predicted values, and as z-scores 39]. The test took place along a flat, straight corridor with a hard surface, rarely travelled by others (40 meters long, marked every meter with cones to specify turnaround points). Patients were instructed to walk as far as possible for 6 minutes, with the option to rest if necessary. Throughout the 6MWT, various parameters were determined at rest (_Rest_) and at the end (_End_) of the walk, including HR (bpm), oxy-haemoglobin saturation (SpO_2_, %), 6MWD (m, % of predicted value), and the number of stops. Blood pressure was measured only at rest to verify the absence of 6MWT contraindication [22, 39, 43]. For some 6MWT parameters, delta exercise changes (Δ_Exercise_ = 6MWT_End_ value minus 6MWT_Rest_ value) were calculated (e.g. Δ_SpO2_, Δ_HR_). The instructions given to the patients during the test were in accordance with international guidelines [22, 39]. HR was expressed as absolute value and as a percentage of the predicted maximal HR (PMHR) (PMHR (bpm) = 208 – (0.7 × age)) [53]. HR and SpO_2_ were measured using a finger pulse oximeter (Beurer PO 40, Shanghai, China). The HR_End_ (bpm) was considered as an HR target for lower limb exercise training [54]. The predicted 6MWD and its lower limit of normal (LLN) were calculated based on North African norms for adults aged 18–40 years [55] and more than 40 years [56]. An abnormal 6MWD was identified when the 6MWD was lower than the LLN [55, 56]. The 6-min walk work (6MWW) (i.e. the product of 6MWD and weight [57]), reflecting the work of walking, was calculated. Clinically significant desaturation was defined as Δ_SpO2_ greater than five points [58].

### Third phase (6 weeks): Exercise training

The constituents of the CPRP were ‘derived’ from preceding national and international recommendations for COVID-19 patients [8, 49, 59–66].

Patients performed exercise training or sedentary activities (depending on randomized allocation to the study groups), and could not be combined with other physical therapy or sports physical activity. The exercise-training phase, exclusive to the IG, was conducted in the morning in two groups of 10–12 patients. This phase comprised 18 sessions (i.e. three sessions/week for six weeks), with each session lasting 60–90 minutes [59]. Patients of the CG were asked to maintain their usual level of sedentary physical activities [26].

**FIG. 3:**
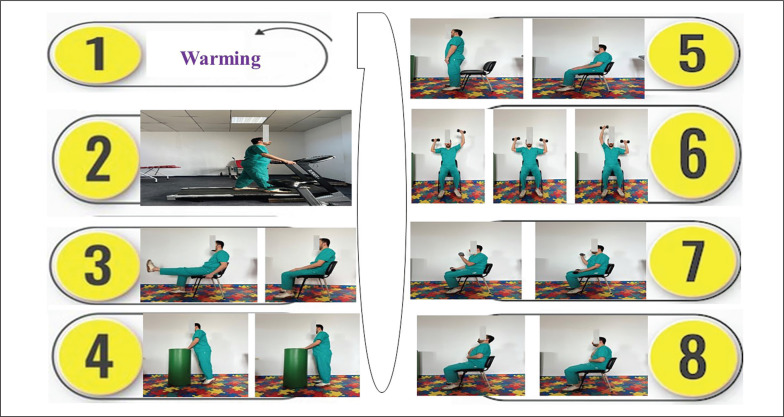
Description of an exercise training session. 1: Warming (5 minutes); 2: Treadmill walking exercise; 3: Knee extension exercise; 4: Heel raises exercise; 5: Sit-stand-sit exercise; 6: Overhead press exercise; 7: Biceps curls exercise; 8: Controlled breathing exercise.

A typical exercise-training session included four parts (Figure 3): *i)* Warming-up for five minutes; *ii)* Aerobic training for 10–35 minutes; *iii)* Resistance training for 15–20 minutes; and *iv)* Respiratory exercises for 15 minutes. During the warm-up phase, light exercises were performed such as walking slowly, mobilization of cervical, lumbar spine, and peripheral joints. In the aerobic-training phase, patients engaged in treadmill walking exercise for a gradually increased duration (e.g. starting with 10 minutes in the first week and increasing by five minutes each week, reaching a total of 35 minutes in the sixth week). Patients were instructed to interrupt the walk if they experienced shortness of breath or dizziness. The walking intensity was personalized using a HR monitor, and the HR target was the HR_End_ ± 5 bpm determined at the end of the 6MWT [37]. On days without aerobic training, patients were advised to perform outdoor walking exercise for the same duration. The strength-training phase involved five types of exercises (Box 1). During the respiratory exercises phase, patients performed controlled breathing and chest expansion exercises. In the sitting position, the patient put one hand on the chest and the other on the abdomen, and performed slow and ample inspiration followed by controlled and maximal expiration.

BOX 1 Description of the 5 types of exercises performed during the strength-training phase of the cardiopulmonary rehabilitation program (CPRP).

**Table:** 

N	Type of exercise	Description
**1**	**Leg extension exercise**	The patient, while sitting on a chair, performed knee extension, and maintained the extension for 5 seconds and returned gradually to the resting position for each repetition. During the first 2 weeks, the 2^nd^ and 3^rd^ weeks, and the 5^th^ and 6^th^ weeks of the CPRP, 1 series of 10 repetitions, 2-series of 10 repetitions, and 3-series of 10 repetitions for each side were performed, respectively.

**2**	**Heel raises exercise**	The patient, while standing up, performed gradual maximal plantar flexion, maintained the contraction for 5 seconds and gradually returned to the resting position During the 1^st^ 2-weeks, 3^rd^ week, and 5^th^ and 6^th^ weeks of the CPRP, 1 series of 10 repetitions, 2-series of 10 repetitions and 2-series of 10 repetitions for each side were performed, respectively.

**3**	**Sit-stand-sit exercise**	The patient started from the seated position and performed controlled stand-ups and returned to the seated position as slowly and as controlled as possible. During the 1^st^ two weeks, the 2^nd^ and 3^rd^ weeks, and the 5^th^ and 6^th^ weeks of the CPRP, 1 series of 10 repetitions, 2-series of 10 repetitions, and 3-series of 10 repetitions for each side were performed, respectively.

**4**	**Overhead press exercise**	The patient performed three-series of 10 repetitions of the movement for each side. Progression in load was obtained through a gradual increase in dumbbell weight

**5**	**Biceps curls exercise**	The patient performed 10 repetitions of alternated elbow flexions while carrying a dumbbell in each hand for three series. Progression in load was obtained through a gradual increase in dumbbell weight.

Throughout each exercise-training part, therapeutic education was provided to enhance patients’ adherence to lifestyle counselling provided during the first phase of the CPRP. This included guidance on managing comorbidities (when applicable), psychological support, and nutritional counselling [49].

### Fourth phase (2 weeks): Post-CPRP evaluations

In this phase, both groups underwent similar evaluations/tests to those conducted in the second phase. The results of the CPRP were communicated, and explained to the patients.

### Data presentation and statistical analyses Expression mode of data

Quantitative and categorical data were presented as mean ± SD (95% confidence interval) and number (%), respectively. For each set of quantitative data (i.e. dyspnoea (mMRC and Borg scales), weight, BMI, spirometric data, 6MWD, 6MWW, HR, SpO_2_, and Δ_Exercise_), changes induced by the CPRP were calculated (Δ_CPRP_ = pre- minus post-CPRP).

### Session effect: Pre- vs. post-CPRP

The Wilcoxon test and the two-sided chi-squared test were used to compare the quantitative and categorical data of the same group determined before and after CPRP, respectively.

### Group effect: IG vs. CG

The Student’s t-test and the two-sided chi-squared test were used to compare the quantitative and categorical data of the two groups before and after CPRP, respectively.

### Group and session effect: Group (IG or CG) vs. session (pre- or post-CPRP)

Analysis of variance (ANOVA) was used to examine the intervention differences (i.e. groups vs. sessions) and to analyse the differences between and within the sessions study and groups. The partial eta-squared effect size was calculated, and Hedge’s values were used for effect size measurement [67]. An effect size of ≤ 0.2 was described as a small effect, around 0.5 as a medium effect, around 0.8 as a large effect, and more than 1.30 as very large effect [67].

### Clinical significant approach

The CPRP was considered ‘efficient’ if the mean Δ_CPRP_ for 6MWD exceeded the recommended MCID of 30 m for 6MWD [39]. A decrease of more than one point in the mean Δ_CPRP_ for mMRC dyspnoea signified a perceived clinical improvement [40, 41].

All statistical procedures were performed using statistical software (StatSoft, Inc. (2011). STATISTICA, version 12). The significance level was set at p < 0.05.

## RESULTS

Among the 45 LC19Ps recruited, 36 agreed to participate in the study (Figure 1). However, only 30 (20 in the IG, 10 in the CG) completed the protocol.

### Patients’ characteristics

The two groups were matched for sex, anthropometric data, comorbidities, and dyspnoea levels before and during COVID-19, COVID-19 data, and computed tomography scan data (Table 1). The only difference was in the consumption of cigarettes (in packyears): the CG had a higher consumption compared to the IG (Table 1).

**TABLE 1:** Characteristics of the 2 groups: Intervention group (IG, n = 20) and control group (CG, n = 10).

Data	Unit/Category	IG (n = 20)	CG (n = 10)	p-value
**Sex and initial anthropometric data**				
Sex	Male	10 (50)	4 (40)	0.604
Age	Year	53 ± 14	52 ± 14	0.922
Height	cm	167 ± 10	167 ± 6	0.919
Weight	kg	79 ± 15	85 ± 26	0.438
Body mass index	kg/m^2^	28.5 ± 4.8	30.2 ± 7.6	0.477
Corpulence status	Underweight	1 (5)	0 (0)	0.472
	Normal weight	2 (10)	2 (20)	0.447
	Overweight	11 (55)	4 (40)	0.439
	Obesity	6 (30)	4 (40)	0.583

**Smoking data and socioeconomic data**				
Smoking cigarette status	Non-smoker	9 (45)	4 (40)	0.794
	Passive smoker	3 (15)	2 (20)	0.729
	Ex-smoker	8 (40)	4 (40)	-
Pack-year		22 ± 8	34 ± 5	0.027^*^
Smoking narghile status	Ex-smoker	4 (20)	1 (10)	0.488
Socioeconomic level	Low	13 (65)	7 (70)	0.784
**Comorbidities**				
Diabetes mellitus	Yes	5 (25)	1 (10)	0.760
Arterial hypertension	Yes	4 (20)	2 (20)	-
Dyslipidemia	Yes	2 (10)	2 (20)	0.447
Dysthyroid	Yes	2 (10)	1 (10)	-

**Dyspnea** (mMRC) **level before and during the COVID-19**				
Before COVID-19	Yes	2 (10)	2 (20)	0.447
	Level-1	18 (90)	8 (80)	0.447
	Level-2	2 (10)	2 (20)	0.447
During COVID-19	Yes	20 (100)	10 (100)	-
	Level-2	3 (15)	2 (20)	0.729
	Level-3	15 (75)	8 (80)	0.760
	Level-4	2 (10)	0 (0)	0.301
Worsening of dyspnea	Yes	20 (100)	10 (100)	-

**COVID-19 data**				
Hospitalization	Yes	11 (55)	5 (50)	0.796
	IUC	5 (25)	1 (10)	0.333
Length of hospitalization	Days	14 ± 9	20 ± 7	0.206
Duration of symptoms	Days	25 ± 24	21 ± 8	0.635
Corticosteroid therapy	Yes	15 (75)	8 (80)	0.760
Oxygen therapy	Yes	14 (70)	6 (60)	0.583
**Computed tomography (CT) scan data**				
CT	Yes	11 (55)	8 (80)	0.180
Extent of lesions	0–10%	1 (5)	1 (10)	0.605
	10–25%	1 (5)	2 (20)	0.197
	25–50%	5 (25)	5 (50)	0.171
	50–75%	4 (20)	0 (0)	0.129

**COVID-19**: Coronavirus disease 2019. **mMRC**: Modified medical research council.

Quantitative and categorical data were mean ± standard deviation and number (%), respectively.

* p-value < 0.05 (Student T-test or two-sided chi-2 test): IG vs. CG.

### Effect on weight, BMI, and corpulence status

ANOVA revealed that the CPRP did not influence weight or BMI. In the IG, the percentage of patients with overweight decreased significantly from 55% to 25% (Table 2).

**TABLE 2:** Impact of the cardiopulmonary rehabilitation program (CPRP) on weight, body mass index (BMI), and dyspnea of the 2 groups: Intervention group (IG, n = 20) and control group (CG, n = 10).

Data	Unit/category	IG (n = 20)			CG (n = 10)			Comparison between 2 groups: p-value			ANOVA: Group vs. Session	

		Pre	Post	Δ	Pre	Post	Δ	Pre- vs. Pre-	Post- vs. Post-	Δ vs. Δ	p-value	Partial eta-squared (effect size)
**Weight**	kg	79 ± 15	81 ± 16	2 ± 13	85 ± 26	85 ± 25	-1 ± 4	0.438	0.623	0.618	0.843	0.001

**BMI**	kg/m^2^	28.5 ± 4.8	29.2 ± 6.1	0.7 ± 4.4	30.2 ± 7.6	30.0 ± 7.5	-0.2 ± 1.4	0.477	0.756	0.562	0.805	0.001

**Corpulence status**	Underweight	1 (5)	0 (0)	-1	0 (0)	0 (0)	0	0.472	-	-	-	-
	Normal weight	2 (10)	5 (25)	+3	2 (20)	3 (30)	-1	0.447	0.773	-	-	-
	Overweight	11 (55)	5 (25)	-6^μ^	4 (40)	3 (30)	-1	0.439	0.773	-	-	-
	Obesity	6 (30)	10 (50)	+4	4 (40)	4 (40)	0	0.583	0.605	-	-	-

**Dyspnea (rest)**	Borg scale	5.2 ± 2.0	1.8 ± 0.9	-3.5 ± 2.0*	5.1 ± 2.0	3.8 ± 1.0	-1.3 ± 1.5	0.896	0.001	0.005	0.010	0.105
	mMRC total	3.0 ± 0.5	1.5 ± 0.6	-1.5 ± 0.8*	2.7 ± 0.5	2.6 ± 0.7	-0.1 ± 0.3	0.208	0.001	0.001	0.007	0.248

**mMRC dyspnea**	Level-1	0 (0)	11 (55)	+11^μ^	0 (0)	1 (10)	+1	-	0.018	-	-	-
	Level-2	3 (15)	8 (40)	+5^μ^	3 (30)	2 (20)	-1	0.333	0.273	-	-	-
	Level-3	15 (75)	1 (5)	-14^μ^	7 (70)	7 (70)	0	0.770	0.001	-	-	-
	Level-4	2 (10)	0 (0)	-2	0 (0)	0 (0)	0	0.301	-	-	-	-

**BMI**: Body mass index. m**MRC**: Modified medical research council. **Post**: After CPRP. **Pre**: Before CPRP. Δ: Post minus Pre. Quantitative and categorical data were mean ± standard deviation, and number (%) respectively.

**Session effect**: Pre vs. Post; *p-value < 0.05 (Wilcoxon test): Quantitative data; μp-value < 0.05 (One-sided chi-2 test): Corpulence status; **Group effect**: IG vs. CG; #p-value < 0.05 (Student test): Pre vs. Pre or Post vs. Post or Δ vs. Δ; θp-value < 0.05 (Two-sided chi-2 test): Pre vs. Pre or Post vs. Post; **Session and group effect** Ψp-value < 0.05 (Analysis of variance: ANOVA).

### Effect on dyspnoea

ANOVA revealed that the CPRP impacted dyspnoea evaluated via the Borg and mMRC scales (Table 2). Compared to the CG, the IG had larger decreases in Δ_CPRP_ for Borg and mMRC scales (-1.3 ± 1.5 vs. -3.5 ± 2.0; and -0.1 ± 0.3 vs. -1.5 ± 0.8; respectively). In the IG, the 1.5-point decrease in mMRC exceeded the recommended MCID of 1 point. The percentage of patients with dyspnoea mMRC level 3 in the IG decreased significantly from 75% to 5%.

### Effect on spirometric data

ANOVA revealed that the CPRP had no impact on spirometric data. In the IG, from pre- to post- CPRP, FEV_1_ and FVC increased by 200 ml (7%) and 180 ml (5%), respectively (Table 3).

**TABLE 3:** Impact of the cardiopulmonary rehabilitation program (CPRP) on spirometric data of the 2 groups: Intervention group (IG, n = 20) and control group (CG, n = 10).

Data	Unit/category	IG (n = 20)			CG (n = 10)			Comparison between 2 groups: p-value			ANOVA: Group vs. Session	

		Pre	Post	Δ	Pre	Post	Δ	Pre- vs. Pre-	Post- vs. Post-	Δ vs. Δ	p-value	Partial eta-squared (effect size)
**FEV_1_**	l	2.56 ± 0.96	2.76 ± 1.01	0.20 ± 0.32*	2.33 ± 0.72	2.34 ± 0.67	0.01 ± 0.13	0.521	0.244	0.075	0.690	0.002
	% predicted	82 ± 24	89 ± 25	7 ± 12*	78 ± 25	78 ± 25	1 ± 4	0.626	0.272	0.119	0.651	0.003
	z-score	-1.23 ± 1.63	-0.77 ± 1.72	0.46 ± 0.74*	-1.56 ± 1.57	-1.52 ± 1.59	0.04 ± 0.28	0.597	0.258	0.099	0.644	0.003

**FVC**	l	3.13 ± 1.12	3.31 ± 1.08	0.18 ± 0.37*	2.96 ± 0.70	2.98 ± 0.74	0.01 ± 0.14	0.674	0.398	0.192	0.768	0.001
	% predicted	80 ± 21	85 ± 21	5 ± 11	79 ± 21	79 ± 23	1 ± 4	0.873	0.494	0.223	0.703	0.002
	z-score	-1.48 ± 1.55	-1.13 ± 1.50	0.35 ± 0.75	-1.60 ± 1.54	-1.54 ± 1.65	0.05 ± 0.30	0.846	0.492	0.236	0.725	0.002

**FEV_1_/FVC**	Absolute value	0.82 ± 0.10	0.83 ± 0.07	0.01 ± 0.08	0.77 ± 0.13	0.78 ± 0.10	0.00 ± 0.07	0.305	0.117	0.847	0.915	0.001
	% predicted	102 ± 12	104 ± 9	1 ± 10	97 ± 17	97 ± 13	1 ± 8	0.297	0.124	0.867	0.925	0.001
	z-score	0.37 ± 1.45	0.51 ± 1.10	0.14 ± 1.2	-0.25 ± 1.60	-0.27 ± 1.42	-0.02 ± 0.90	0.297	0.112	0.726	0.837	0.001

**FEV_1_**: Forced expiratory volume in one second. **FVC**: Forced vital capacity. **Post**: After CPRP. **Pre**: Before CPRP. Δ: Post minus Pre. Data were mean ± standard deviation.

**Session effect**: Pre vs. Post; *p-value < 0.05 (Wilcoxon test): Quantitative data. **Group effect**: IG vs. CG; #p-value < 0.05 (Student test): Pre vs. Pre or Post vs. Post or Δ vs. Δ. **Session and group effect**: Ψp-value < 0.05 (Analysis of variance: ANOVA).

### Effect on submaximal exercise data

No patient stopped during the 6MWT, and no side effects were noted.

ANOVA revealed that the CPRP impacted the 6MWD (m, %) and the HR_rest_ (bmp, %), but did not impact the 6MWW and SpO_2_ (Table 4). First, compared to the CG, the IG had a higher Δ_CPRP_ for 6MWD (m, %) (5 ± 45 vs.168 ± 99; and 1 ± 8 vs. 28 ± 8; respectively), with small effect sizes. In the IG, the 168 m Δ_CPRP_ for 6MWD greatly exceeded the recommended MCID of 30 m. Second, compared to the CG, the IG had a higher Δ_CPRP_ for HR_rest_ (bpm, %) (1 ± 7 vs. -9 ± 9; and 0 ± 4 vs. -5 ± 6; respectively). In the IG, the percentages of patients with abnormal 6MWD or desaturation during the 6MWT decreased from 100% to 75%, and from 30% to 10%, respectively.

**TABLE 4:** Impact of the cardiopulmonary rehabilitation program (CPRP) on submaximal exercise data of the 2 groups: Intervention group (IG, n = 20) and control group (CG, n = 10).

Data	Unit/category	IG (n = 20)			CG (n = 10)			Comparison between 2 groups: p-value			ANOVA: Group vs. Session	

		Pre	Post	Δ	Pre	Post	Δ	Pre- vs. Pre-	Post- vs. Post-	Δ vs. Δ	p-value	Partial eta-squared (effect size)
**6MWD**	m	349 ± 137	517 ± 115	168 ± 99*	414 ± 106	419 ± 78	5 ± 45	0.194	0.023	0.001	0.013	0.104

	% predicted	53 ± 16	81 ± 14	28 ± 8*	67 ± 18	68 ± 14	1 ± 8	0.044	0.017	0.001	0.002	0.157

	< LLN	20 (100)	13 (75)	-7^μ^	8 (80)	8 (80)	0	0.038	0.760	-	-	-

**6MWW**	mkg	27383 ± 11455	41536 ± 10859	14153 ± 11096*	37226 ± 22018	36847 ± 18757	-379 ± 4577	0.116	0.391	0.001	0.078	0.054

HR (bpm)	Rest	84 ± 9	75 ± 4	-9 ± 9*	81 ± 7	82 ± 6	1 ± 7	0.367	0.001	0.007	0.014	0.102

	End	106 ± 10	96 ± 6	-10 ± 13*	105 ± 11	95 ± 7	-10 ± 8*	0.782	0.729	0.965	0.966	0.001

	**ΔWalk**	21 ± 12	21 ± 8	0 ± 15	23 ± 11	13 ± 9	-10 ± 10*	0.653	0.034	0.076	0.091	0.050

**HR (%)**	Rest	49 ± 6	44 ± 3	-5 ± 6*	48 ± 5	48 ± 5	0 ± 4	0.425	0.012	0.007	0.041	0.072

	End	62 ± 7	56 ± 6	-6 ± 8*	61 ± 8	56 ± 5	-6 ± 4*	0.781	0.738	0.983	0.987	0.001

	ΔWalk	12 ± 7	12 ± 5	0 ± 9	14 ± 6	8 ± 5	-6 ± 6	0.662	0.032	0.069	0.087	0.051

**SpO_2_ (%)**	Rest	96 ± 2	98 ± 1	2 ± 2*	97 ± 1	96 ± 1	0 ± 1	0.635	0.005	0.007	0.056	0.063

	End	93 ± 3	96 ± 3	2 ± 3*	94 ± 2	94 ± 2	0 ± 3	0.523	0.141	0.112	0.153	0.036

	**ΔWalk**	-3 ± 3	-2 ± 2	1 ± 3	-2 ± 2	-2 ± 2	0 ± 3	0.710	0.894	0.694	0.702	0.003

	**Desaturation**	6 (30)	2 (10)	-4^μ^	1 (10)	2 (20)	+1	0.222	0.038	-	-	-

**HR**: Heart-rate. **LLN**: Lower limit of normal. _**End**_: End of walk. **Post**: After CPRP. **Pre**: Before CPRP. _**Rest**_: Before the walk. **SpO_2_**: Oxyhemoglobin saturation. **6MWD**: 6-min walk distance. **6MWW**: 6-min walk work. Δ: Post minus Pre. Δ_**Walk**_: _End_ minus Rest. Quantitative and categorical data were mean ± standard deviation (95% confidence interval) and number (%), respectively. **Session effect**: Pre vs. Post; *p-value < 0.05 (Wilcoxon test): Quantitative data; μp-value < 0.05 (One-sided chi-2 test): 6MWD < LLN or desaturation. **Groups effect: IG vs. CG** ^#^p-value < 0.05 (Student test): Pre vs. Pre or Post vs. Post or Δ vs. Δ; ^θ^p-value < 0.05 (Two-sided chi-2 test): Pre vs. Pre or Post vs. Post. **Session and group effect**: ^Ψ^p-value < 0.05 (Analysis of variance: ANOVA).

## DISCUSSION

Our Tunisian RCT, investigating the effects of a CPRP on submaximal aerobic capacity, dyspnoea and spirometric data in LC19Ps, revealed that compared to the CG, the IG demonstrated statistically significantly higher changes in *i)* 6MWD (m, %) (5 ± 45 vs. 168 ± 99; 1 ± 8 vs. 28 ± 8; respectively) and HR_rest_ (bpm, %) (1 ± 7 vs. -9 ± 9; 0 ± 4 vs. -5 ± 6; respectively), with small effect sizes. The IG’s 168 m Δ_CPRP_ for 6MWD significantly exceeded the recommended MCID of 30 m; and *ii)* Borg and mMRC scales (-1.3 ± 1.5 vs. -3.5 ± 2.0; and -0.1 ± 0.3 vs. -1.5 ± 0.8, respectively). The IG’s 1.5-point decrease in mMRC significantly exceeded the recommended MCID of 1 point. The two groups exhibited comparable changes in spirometric data.

Our results contribute valuable insights to the growing body of literature on the efficacy of CPRPs for LC19Ps, emphasizing improvements in aerobic capacity and dyspnoea. To the best of the authors’ knowledge, up until December 30, 2023, only 11 RCTs had assessed the effects of a CPRP on the submaximal aerobic capacity of LC19Ps [26–36]. Table 1S to Table 6S in the Appendix provide insights into the methodological characteristics (Table 1S), recruitment methods, inclusion, non-inclusion and exclusion criteria (Table 2S), patient characteristics (Table 3S), 6MWT methodological aspects (Table 4S), CPRP details (Table 5S), and the effects of CPRPs on 6MWT (Table 6S) across these studies. This RCT stands out as the second North-African study in this domain, with the first being conducted in Egypt [29].

### Rationale for choosing the 6MWD data as the main outcome

The assessment of exercise tolerance is traditionally conducted by measuring maximum oxygen consumption in a cardiorespiratory test [68]. However, this approach necessitates sophisticated and expensive equipment, along with personnel possessing advanced skills for its operation [68]. Consequently, the repeated use of such tests poses a substantial financial burden and is not practically feasible on a large scale [68]. Recognizing these challenges, simpler assessments such as the 6MWT have gained popularity [22, 39, 43]. The latter entails measuring the distance an individual can walk on a flat surface within a span of 6 minutes [22, 39, 43]. The 6MWT comes with various benefits, presented in detail in the Appendix. Its simplicity, ease of administration, and minimal resource requirements make it a practical choice for assessing exercise tolerance in various settings and populations [22, 39, 43]. In comparison to complex cardiorespiratory tests, the 6MWT strikes a balance between providing meaningful insights into functional exercise capacity and addressing the logistical constraints associated with assessments that are more sophisticated.

### Effect on 6MWD

In our RCT, both statistical and clinical significance of the increase in the 6MWD were observed. Compared to the CG, the IG demonstrated a higher Δ_CPRP_ for 6MWD (m, %) (5 ± 45 vs. 168 ± 99; 1 ± 8 vs. 28 ± 8, respectively), albeit with small effect sizes.

Regarding the 6MWD expressed in absolute value (i.e., m), our results are consistent with findings from related two RCTs [29, 30]. A significant group-session effect (i.e. comparison of Δ_CPRP_) was reported [29, 30], indicating a higher ΔCPRP for 6MWD in the IG compared to the CG (57 ± 48 vs. 17 ± 10 [29], 54 vs. 5 [30], respectively) (Table 6S). Among the remaining nine related RCTs (Table 6S), three failed to report the group-session effect [26–28], and six reported no group-session effect [31–36]. In contrast to our RCT, where the effect size for 6MWD was small, one RCT [30] reported a large effect size. Between two RCTs [34, 35] that reported no group-session effect, the effect sizes for 6MWD were small.

Regarding the 6MWD expressed as a percentage of predicted values (i.e. %), the only RCT that chose this expression mode [28] failed to report the group-session effect (Table 6S). However, in contrast to our findings (Table 4), this particular RCT [28] reported no group – or session – effects, as the 6MWD values measured before and after CPRP in both the CG and the IG were comparable (92 ± 14 vs. 96 ± 16%, and 86 ± 17 vs. 89 ± 13%, respectively (Table 6S)). In practice, it is crucial to interpret the 6MWD by comparing it to normal values [22, 39, 43]. The latter are essential to guide the diagnostic and prognostic use of the 6MWT [22, 43]. The success in medical decision-making depends as much on selecting and properly using norms and their limits [22, 43].

In our RCT, the Δ_CPRP_ for 6MWD in the IG and CG were 168 m (i.e. > 30 m MCID [39]) and 5 m (i.e. < 30 m MCID [39]), respectively (Table 4). First, two relevant RCTs [27, 32] adopted the MCID approach for 6MWD, setting it at 50 m (Table 4S). Consistent with our RCT, these two RCTs reported statistically significant effects, as the 6MWD in the IGs were 85 [27] and 95 [32] m (Table 6S). However, in one RCT [27], the 6MWD change in the CG was below the 50 m MCID (i.e. 15 m), while in the other RCT [32], it significantly exceeded the 50-m MCID (i.e., 72 m) (Table 6S). Second, the 6MWD change observed in the IG of the remaining RCTs surpassed the recommended MCID of 30 m [39] (63 [33], 57 [29, 34, 35], 54 [30] m) (Table 6S). Third, the 6MWD change observed in the CG of the remaining RCTs either exceeded the recommended MCID of 30 m [39] in three RCTs [57 [33], 39 [34, 35] m], or fell below the recommended MCID of 30 m [39] in two RCTs [5 [30], 17 [29] m)] (Table 6S). The considerable variation in 6MWD changes observed in our RCT and the 11 other RCTs highlights the necessity of establishing the 6MWD MCID in LC19Ps [25]. The aforementioned findings from our RCT and the 11 other RCTs (Table 6S) align with those reported by previous SRs [25, 69–74], confirming the effectiveness of CPRPs in improving various health outcomes, including physical health, in LC19Ps. The results of the aforementioned seven SRs [25, 69–74] are extensively detailed in the Appendix.

The percentage of patients exhibiting an abnormal 6MWD remained steady at 80% in the CG, while it decreased from 100% (pre-CPRP) to 75% (post-CPRP) in the IG (Table 4). First, no prior related RCT has compared the percentage of LC19Ps or cases with abnormal 6MWD before and after CPRP. Second, our results contrast with those reported in a German study [15], where 79% of mild/moderate COVID-19 patients exhibited an abnormal 6MWD after three weeks of inpatient CPRP. Third, a previous observational study indicated that the percentages of LC19Ps with abnormal 6MWD decreased from 21% to 0% [37].

The two groups in our RCT showed comparable changes in 6MWW, but in the IG, the 6MWW change increased by 14,153 mkg (Table 4). First, no previous related RCT has assessed the 6MWW, which may offer a more comprehensive estimation of the work required to perform the test compared to 6MWD alone [37]. Second, in agreement with our findings, a prior observational study indicated that the 6MWW change in LC19Ps increased by 2448 mkg [37]. Since weight directly influences the energy needed for completing the 6MWT [22, 39], future related RCTs should consider calculating and reporting the 6MWW, as it can provide valuable insights into patients’ functional capacity [25].

### Effect on dyspnoea

In comparison to the CG, the IG demonstrated higher changes in Borg and mMRC scales (-1.3 ± 1.5 vs. -3.5 ± 2.0; and -0.1 ± 0.3 vs. -1.5 ± 0.8, respectively). In related RCTs (Table 4S), conflicting dyspnoea values were reported in eight studies [27, 29–35]. On one hand, four related RCTs reported effects of CPRP on mMRC [29–31, 33], while two others [27, 32] reported no effects. For instance, in one RCT [29], the mean mMRC between-group differences decreased by 49%, from a score of 2.63 ± 0.60 to 1.38 ± 0.49. On the other hand, while one RCT reported significant effects of CPRP on the Borg scale [31], two others [34, 35] reported no effects. Some previous SRs [24, 69, 70, 72, 73], described in detail in the Appendix, reported conflicting findings related to the effects of CPRP on dyspnoea. Previous studies have indicated that CPRP improves perceived dyspnoea_Rest_, regardless of the mode of evaluation (e.g. mMRC [15, 18], chronic obstructive pulmonary disease assessment test [16]), even in severe/critical COVID19 patients [15].

In our IG, the percentage of patients experiencing dyspnoea at mMRC level 3 decreased significantly from 75% to 5%, and the 1.5-point decrease in mMRC exceeded the recommended MCID of 1 point [40, 41]. This aligns with the findings of one observational study [37], where mMRC improvement surpassed than the 1-point MCID [40].

Dyspnoea is a crucial determinant of the 6MWD in patients with chronic respiratory disease, reflecting both the physiology of exercise limitation and the impact of exercise limitation on daily life [22, 39]. However, comparing dyspnoea data between studies with different assessment scales (Table 4S) is challenging due to the lack of standardization and potential scale-related variations in measurement [25]. The observed reduction in dyspnoea perception during CPRP might be attributed to physiological adaption to exercise training [75]. The reduction of dyspnoea, one of the most common symptoms among individuals with chronic respiratory disease, is an important target of CPRPs [76]. In LC19Ps, the improvement in perceived dyspnoea is crucial, as dyspnoea is significantly linked with a higher mortality [77], and serves as a predictive factor of reduced functional capacity [78].

### Effect on HR

Regarding HR expressed in bpm, our RCT revealed that: *i)* compared to the CG, the IG had a larger change in HR_rest_ (1 ± 7 vs. -9 ± 9; respectively), with a small effect size; *ii)* compared to before CPRP, after CPRP both HR_Rest_ and HR_End_ of the IG decreased by 9 ± 9 and 10 ± 13, respectively; and *iii)* compared to before CPRP, after CPRP HR_Rest_ in the CG remained unchanged, but HR_End_ decreased by 10 ± 8 (Table 4). Concerning HR expressed as a percentage of PMHR, our RCT identified that: *i)* compared to the CG, the IG had a larger change in HR_Rest_ (0 ± 4 vs. -5 ± 6, respectively), with a small effect size, *ii)* compared to before CPRP, after CPRP both HR_Rest_ and HR_End_ of the IG decreased by 5 ± 6 and 6 ± 8, respectively; and *iii)* compared to before CPRP, after CPRP HR_Rest_ of the CG remained unchanged, but HR_End_ decreased significantly by 6 ± 4 (Table 4). During the CPRP, expressing HR as a percentage of PMHR accommodates individual variations in fitness levels and age, allowing for personalized exercise intensity assessment [37]. Among the related 11 RCTs (Table 4S), HR responses during the 6MWT were measured in three RCTs [26–28], but only one reported HR values in bpm and not as a percentage of PMHR [28]. This particular RCT identified that *i)* HR_Rest_ was comparable in IG and CG both pre- (84 ± 12 vs. 78 ± 14, respectively) and post- (82 ± 10 vs. 78 ± 14, respectively) CPRP; and *ii)* HR_End_ was higher in the IG than the CG both before (118 ± 18 vs. 100 ± 14, respectively) and after (118 ± 19 vs. 106 ± 14, respectively) CPRP [28].

An increased HR_Rest_, even after adjustment for fitness, serves as an independent risk factor for all-cause mortality [79], and a 10-bpm increase in HR_Rest_ may elevate all-cause mortality by 17% [80]. An observational study [37] reported a post-CPRP decrease in HR_Rest_ by 7 ± 9 bpm (5 ± 5%). The authors suggested that their finding indicates a beneficial effect for endurance-based exercise training as well as combined exercise training [37]. In our RCT, the observed decrease in HR_Rest_ reflects the positive impact of endurance and combined exercises [81], and could be attributed to improvements in physical fitness [82], sleep quality [83] and diet [39]. Monitoring HR during the 6MWT proves valuable, offering insights into exercise performance and cardiovascular responses, and therefore, should be included in 6MWT assessments [22, 39]. HR responses may contribute to the performance of the 6MWT in patients with chronic respiratory disease, and HR patterns (e.g. HR_Rest_, HR_End_, maximum HR, increase in HR, and HR recovery at two minutes) can be among the factors explaining a part of the improvement in the 6MWD [22, 39].

### Effect on SpO_2_

Our CPRP did not influence SpO_2_ (Table 4). First, among the 11 RCTs, SpO_2_ responses during the 6MWT were measured in three RCTs [26–28], but only one [28] reported SpO_2_ values. This particular RCT identified that both groups increased SpO_2End_ after CPRP, while pre-CPRP SpO_2_ increased only in the IG [28]. Second, an observational study reported that CPRP had no effect on both SpO_2Rest_ and SpO_2End_ of LC19Ps [37]. The lowest SpO_2_ recorded during a 6MWT, which may not align with SpO_2End_, has emerged as an important marker of disease severity and prognosis [22, 39]. Moreover, oxygen desaturation during a 6MWT provides valuable information regarding exercise-induced desaturation, disease severity, and disease progression [22, 39].

### Effect on spirometric data

ANOVA revealed that our CPRP had no effect on the spirometric data. In the IG, from before to after CPRP, FEV_1_ and FVC increased by 200 ml (7%) and 180 ml (5%), respectively (Table 3). First, the findings of our RCT align with the conclusion of a 2023 SR and meta-analysis aiming to examine the efficacy of CPRP in LC19Ps [84]. The meta-analysis results of the included 10 RCTs reported no significant differences in lung function [84]. For example, the FVC pooled mean difference (MD) showed a non-significant overall effect of CPRP compared to the comparator groups (MD = 0.21). The results show heterogeneity, detecting a significant variability with I2 = 66%, not attributable to chance [84]. Second, the results observed in our IG are comparable to those reported in some studies indicating improvements in FEV_1_ [15, 17, 26] and/or FVC [15, 17, 18, 26]. The 200-ml increase in FEV_1_ observed in our IG is higher than, equal to, and lower than some values reported in the literature (e.g. 110 ml [37], 200 ml [17], 340 ml [26], respectively). On one hand, since there is a positive correlation between FEV_1_ and/or FVC and 6MWD [56], the increases in FEV_1_ and FVC could explain a part of the increase in the 6MWD. On the other hand, these increases are useful to improve risk stratification in patients with intermediate coronary heart disease [85]. The improvement in FVC and FEV_1_ in the IG could be partly explained by the breathing exercises applied during CPRP [15, 17, 26]. However, some authors reported that there is presumably a spontaneous improvement over time of lung function in LC19Ps, and there is a natural upturn occurring without the influence of CPRP [86].

### Effect on weight, BMI, and corpulence status

ANOVA revealed that the CPRP did not impact weight or BMI. In the IG, the percentage of patients with overweight decreased from 55% to 25% (Table 2). Our results contrast with those of one RCT [28], which reported a significant “group vs. session” effect of CPRP on weight. In this particular RCT [28], weight increased by 2.8 ± 0.6 kg in the CG (i.e. pre-CPRP = 89.0 ± 21.7 kg; post-CPRP = 90.5 ± 22.0 kg), whereas a tendency toward increase by 1.9 ± 0.5 kg occurred in the IG (i.e. pre-CPRP = 89.1 ± 14.4 kg; post-CPRP = 90.4 ± 14.1 kg). This difference may be explained by the higher BMI of their patients at baseline and the fact that they included only post-hospitalization patients who may experience an increase in dietary intake after their discharge [28].

### Discussion of the methodology

The disparities observed between our results and those of some related RCTs (Tables 1S to 6S, Appendix) can be attributed to at least 12 factors related to differences in *(i)* recruitment methods, *(ii)* applied questionnaires and tests, *(iii)* blinding technique and randomization method, *(iv)* primary and secondary outcomes, *(v)* time interval between the onset of COVID-19 and the initiation of the CPRP, *(vi)* applied inclusion, non-inclusion and exclusion criteria, *(vii)* sample size calculation, *(viii)* characteristics of LC19Ps and controls, *(ix)* methodological aspects of the 6MWT, *(x)* collected data during the 6MWT, *(xi)* methodological aspects of the CPRP, and *(xii)* statistical approaches. A discussion related to points *(i)* to *(vi)* is detailed in the Appendix, and a discussion related to points *(vii)* to *(xii)* is provided in the following sentences.

### Sample size calculation

Similar to eight related RCTs [26, 27, 29, 30, 32–35] (Table 1S), we calculated the required sample size using a predictive equation [45]. Our sample size (n = 30) exceeded that of one RCT (n = 17) [32], was comparable to the samples of three RCTs (n = 32 [28, 34, 35]), and was smaller than the samples of seven RCTs (n = 42 [33], n = 52 [29, 30], n = 72 [26], n = 81 [36], n = 119 [27], and n = 392 [31]) (Table 3S). Determining the optimal sample size is crucial as it helps avoid inadequate power to detect statistical effects [87], and ensures a representative sample for detecting statistical significance [45]. A large sample size is expensive and exposes more participants to measures [87], but using an insufficient number of participants may result in lower precision in the results.

### Characteristics of LC19Ps and controls

The characteristics of our patients and controls, including the ratio between the CG and IG, sex, age, corpulence status, comorbidities, COVID-19 data, and smoking status, fall within intermediate ranges compared to related similar studies (Table 3S). First, as in some related RCTs [34–36], our sample size distribution for the CG and IG was lower than one. In the remaining RCTs, the sample size distributions were either “equal” [26, 27, 29–33], or higher than [28] ours. Second, similar to some related RCTs [26–28, 30–35], we included both sexes. Third, the mean age of our patients (i.e. 52–53 years) was intermediate compared to that reported in the literature: higher than in some RCTs [29, 31], comparable to others [27, 28, 30, 32], and lower than in some RCTS [26, 33–36] (Table 3S). Fourth, the observed frequency of obesity in our RCT (i.e. 33%, Table 1) was lower than in one RCT (i.e. 65–67% [28]) and higher than in another RCT (i.e. 13–15% [27]). Fifth, the most frequent comorbidities in our RCT, diabetes mellitus, and arterial hypertension (each 20%, Table 1) were consistent with those reported in some related RCTs [26–28, 30, 33] (Table 3S). Sixth, the initial extent of COVID-19 lesions observed in our RCT (Table 1) was comparable to that reported in another related RCT [29] (Table 3S). Finally, the percentages of ex-smokers of cigarettes (40%) or narghile (17%) reported in our RCT (Table 1) are comparable to those reported in the literature, with frequencies varying from 10% [27] to 68% [33] (Table 3S).

### Methodological aspects of the 6MWT

In alignment with four previous RCTs [27, 28, 34, 35], we conducted the 6MWT according to the most updated 6MWT guideline, specifically the European respiratory society (ERS)/American Thoracic Society (ATS)-2014 guideline [22, 39]) (Table 4S). It is noteworthy that five related RCTs [30–33, 36] applied an outdated guideline for the 6MWT (i.e. ATS-2002 guideline [43]), despite updated guidelines being available since 2014. Adhering to the most current guidelines is crucial for ensuring the accuracy and relevance of the test results [25].

We implemented the 6MWT along a 40 m-long corridor, while in related RCTs, corridor lengths varied between 30 [27, 28, 30, 33–36] and 35 [29] m (Table 4S). The length of the corridor can impact performance [22, 43], and guidelines suggest a walking course of at least 30 m [43]. Research indicated that there are no significant differences in outcomes when tracks of lengths ranging from 15 to 50 m are used [88]. Consistency in corridor length is essential for accurate comparisons of 6MWT results [25, 88].

In contrast to two related RCTs [29, 36] where the 6MWT was conducted indoors, we performed it outdoors. Other related RCTs implemented the 6MWT both indoors and outdoors [27, 32] (Table 4S). Guidelines recommend performing the 6MWT indoors [43], but it can also be conducted outdoors if weather conditions permit. Research suggested MDs in 6MWD (i.e. MD of 4 m) between indoor and outdoor courses [89]. This flexibility allows adaptation to different clinical settings and patient comfort [25].

Similar to two related RCTs [34, 35], our patients were allowed to move independently and rest if necessary. In contrast to one related RCT [27], our patients were not allowed to use walking aids during the 6MWT. The use of walking aids during the 6MWT can significantly influence the 6MWD [22, 39]. This information is important for interpreting and comparing results across different studies [25].

### Collected data during the 6MWT

Before the initiation of the 6MWT (i.e., at rest), we assessed dyspnoea using two scales, namely the Borg and mMRC scales. Among the 11 related RCTs (Table 4S), dyspnoea was evaluated in nine [26, 27, 29–35] using the mMRC scale [27, 29, 30, 32], the Borg scale [26, 34, 35], or both mMRC and Borg scales [31, 33].

During the 6MWT, we determined the following parameters: 6MWD, 6MWW, HR, and SpO_2_. First, while all related RCTs determined the 6MWD (Table 4S), only three determined HR and SpO_2_ [26–28], and none reported the 6MWW. Second, in the related RCTs (Table 4S), some studies also measured additional parameters, including SBP and DBP [26–28], respiratory rate [26], fatigue [34, 35], and blood pressure, HR, and SpO_2_ at recovery [28]. Third, akin to all related RCTs (Table 4S), we reported 6MWD as an absolute value, and as performed in one RCT [28], we also expressed it as a percentage of predicted norms. Fourth, mirroring one related RCT [28], we expressed HR in bpm, and as a percentage of PMHR. Finally, in contrast to our study, where desaturation was defined as Δ_SpO2_ > 5 points [58], in one related RCT [28], it was defined as Δ_SpO2_ ≥ 4 points. The latter definition is debatable, as in several studies [56, 58, 90], the most used definition for clinically significant desaturation is Δ_SpO2_ > 5 points. Some studies recommended considering novel desaturation indices, such as the distance-saturation product, desaturation area, and desaturation-distance ratio, for a more comprehensive assessment in future RCTs [22, 39].

### Methodological aspects of the CPRP

The design and execution of CPRPs can significantly impact their outcomes and benefits for patients with chronic respiratory conditions [25]. Four points related to the CPRP merit discussion.

First, our CPRP was conducted at the hospital, aligning with other related RCTs [29, 30, 34–36], and differing from others where CPRPs were administered at home [27, 28, 33] or both at home and at the hospital [26, 27, 32] (Table 5S). The choice of CPRP location, whether at home, at hospital, or a combination of both, substantially influences programme results [25, 91]. Hospital-based CPRPs offer several advantages, such as a structured and supervised environment, access to various exercise equipment, peer support, and expert guidance [25, 91]. In contrast, homebased CPRPs provide more flexibility, convenience, personalized plans, and opportunities for self-management [25, 91]. Decisions on CPRPs’ location should consider patients’ needs, preferences, and desired outcomes [25].

Second, our CPRP comprised three sessions per week for six weeks, with each session lasting 60–90 minutes. The frequency of sessions per week (e.g. one [30, 32], two [26], three [27–29, 32, 33], four [27, 32], five [28, 30, 33–35], six [36], seven [33], 21 [30]), the duration of the CPRP (e.g. two [31], three [34, 35], four [32], five [30], six [26, 27, 29, 36], eight [33], 12 [28] weeks), and session durations (in minutes) (e.g. less than 3–4 [29], 10 [26], 10–30 [28], 20–30 [33], and 40–60 [32]) varied among similar related RCTs (Table 5S). These variances can significantly affect CPRP outcomes [25, 91]. Higher session frequency (e.g. 3–5 sessions/week) improves conditioning, offering consistency and greater social interaction, while lower frequency (e.g., 1–2 sessions/week) provides flexibility, sustainability, and gradual progression [25, 91]. Regarding programme duration, shorter programmes (e.g. 4–8 weeks) focus intensively and suit acute needs, while longer programmes (e.g. ≥12–19 weeks) sustain benefits, induce behavioural changes, and instil patient confidence [25, 91]. The optimal number of sessions/week and CPRP duration should be individualized based on factors such as respiratory condition severity, patients’ baseline fitness levels, goals, and their ability to commit to the programme [25, 91]. The American College of Sports Medicine recommends 16 weeks of supervised aerobic exercise training for inducing training adaptations [92].

Third, our typical exercise-training session included warmingup, aerobic training, resistance training, and respiratory exercises (Figure 3). In related RCTs (Table 5S), different CPRPs were applied in cases including respiratory muscle training [26, 27, 29, 30], breathing exercises [27, 29, 30, 31], bough exercise [26], stretching exercise [26], strength exercises [27], aerobic exercise [27, 28, 30], resistance training [28], and Baduanjin exercise [31]. Variations in CPRP content critically influence its outcomes [25]. A well-designed and comprehensive CPRP can significantly improve the health and well-being of individuals with chronic respiratory conditions [25, 91]. Regarding the CPRP content, a 2023 SR [25] extensively described key elements to be considered. In line with some related RCTs [26], our controls were instructed to maintain sedentary activities. In related RCTs (Table 5S), controls were recommended various activities, including short educational instructions [27], inspiratory muscle training [29], pursed lip breathing, diaphragmatic breathing exercise, and aerobic exercise training [30], and standard rehabilitation treatments such as lip and abdominal breathing training, and respiratory rhythm training [31]. The application of different approaches in controls complicates direct comparisons between RCTs, making it essential to consider CG characteristics when interpreting CPRP outcomes [25].

Fourth, in our RCT, CPRP local guidelines/recommendations and international recommendations [8, 49, 59–66] were applied. In related RCTs (Table 5S), one [27] followed the American College of Sports Medicine guideline [92], and five [30, 31, 34–36] followed guidelines developed based on previous studies. Numerous recommendations/guidelines related to managing LC19Ps exist in the literature [8, 49, 60–66]. These guidelines target specialist physicians such as pulmonologists, cardiologists, physical medicine and rehabilitation specialists, or a combination of different specialties [60, 61, 65, 66], or general practitioners [8, 49, 64]. When comparing RCT results that used different CPRP guidelines, the main challenge is the potential for inconsistency and variability in CPRP design and implementation [25]. This can complicate drawing meaningful conclusions and making direct comparisons between these RCTs [25]. Adherence to evidence-based guidelines can enhance CPRP quality and consistency, improving patient outcomes [25].

### Applied statistical approaches

In our RCT, we employed both statistical and clinical significance approaches. The statistical significant approach, utilized in all related RCTs (Table 4S), typically emphasizes statistical significance to assess differences between groups (i.e. to compare the 6MWD between cases and controls), with a “p value” < 0.05 considered significant. Presently, this approach faces criticism [93]. The clinical significance approach, which was applied in four related RCTs [27, 32, 34, 35] determines whether the results influence medical exercise. It consists in calculating the effect size for some parameters or using the MCID for one specific parameter (e.g. MCID for the 6MWD [27, 32] or dyspnoea). First, as done in two related RCTs [34, 35], we calculated the effect size, which measures the magnitude of the difference between groups or the effect of an intervention [94]. Second, the MCID approach is valuable for assessing whether observed changes in one parameter such as 6MWD or dyspnoea hold practical clinical relevance beyond statistical significance (e.g. the smallest change considered meaningful by patients or clinicians) [95]. In clinical practice, as the MCID for LC19Ps has not been established yet [75], one SR recommended deeming a CPRP ‘efficient’ if the mean changes for 6MWD and dyspnoea (mMRC) exceed recommended MCIDs (i.e. 30 m [39], and one point [40], respectively) [25]. While we set the 6MWD MCID at 30 m (i.e. as per ERS-ATS recommendations [39]), other authors [27, 32] have set it at 50 m. Moreover, following one SR’s recommendation [25], we compared the percentage of LC19Ps or controls with an abnormal 6MWD (i.e. 6MWD < LLN) before and after CPRP. This analysis provides insights into the extent to which CPRPs help patients achieve values within a normal range [37].

### Study strengths and limitations

Our RCT exhibits three notable strengths. First, it was conducted in an outpatient unit in a low-income country, specifically Tunisia. Second, the sample size was calculated, enhancing the study’s statistical robustness [45]. Third, we applied the clinically significant approach, incorporating the MCID of 30 m for the 6MWD [39] and 1 point for mMRC [40].

The present RCT has five limitations. First, we did not measure blood pressure at the end of the 6MWT. Assessing 6MWT_End_ blood pressure is valuable for evaluating cardiovascular health, exercise responses, and overall patient safety [20, 56, 90]. This information can aid in diagnosis, risk assessment, exercise prescription, and patient care in cardiovascular conditions [20, 56, 90]. Notably, high blood pressure is one of the most critical modifiable risk factors for cardiovascular disease and mortality [96]. A related RCT [28] reported that SBP_End_ and DBP_End_ measured before and after CPRP remained unchanged in both cases (SBP (mmHg): 151 ± 24 vs. 158 ± 17, DBP: 90 ± 14 vs. 93 ± 11, respectively) and controls (SBP (mmHg): 147 ± 29 vs. 151 ± 25, DBP: 87 ± 18 vs. 88 ± 16, respectively). Another observational study [37] reported a decrease in post-CPRP DBP_Rest_, with the mean DBP_Rest_ decreasing from 85 to 79 mmHg. This outcome is noteworthy, aligning with the 2018 European Society of Cardiology recommendation of an optimal DBP_Rest_ target between 70 and 80 mmHg for patients across all risk levels [96]. Second, we did not determine SpO_2_ at recovery. Assessing SpO_2_ at recovery provides insights into how effectively a patient’s body returns to a stable SpO_2_ level after exertion [97]. This parameter can reveal the functionality of the cardiovascular and respiratory systems in responding to exercise stress [97]. Third, bronchodilator tests were not performed for our patients. It has been suggested that the bronchodilator response could serve as a predictor of lung function improvement after CPRP in LC19Ps [98]. Fourth, a broader exploration of respiratory function using additional tests, such as plethysmography [17], diffusion capacity of the lungs for carbon monoxide [15, 17, 26], maximal inspiratory pressure [17], and cardiorespiratory tests to determine the ventilatory threshold exercise data using a cardiorespiratory test in order to determine the ventilatory threshold, would have been more comprehensive. In COVID-19 patients, altered diffusing capacity of the lungs for carbon monoxide is a frequent impairment (39%) [99]. Unfortunately, due to the unavailability of equipment in our public health hospital, these additional tests were not conducted. Finally, we did not assess waist circumferences of our patients. Evaluating waist circumference in LC19Ps during a CPRP is important for assessing cardiometabolic health, stratifying risk, monitoring progress, implementing lifestyle interventions, and delivering comprehensive patient care.

## CONCLUSIONS

In summary, the outcomes of our Tunisian RCT demonstrate significant improvements within the IG compared to the CG. Particularly noteworthy are the statistically significant and clinically meaningful increases in the 6MWD and reductions in HR_Rest_ within the IG, surpassing the recommended MCID thresholds. Additionally, participants in the IG experienced significantly greater decreases in Borg and mMRC scales, with the latter achieving a reduction exceeding the MCID. While spirometric data remained comparable between the two groups, the observed positive outcomes in submaximal aerobic capacity and symptomatology underscore the potential efficacy of cardiopulmonary rehabilitation in enhancing the health status of LC19Ps. These findings contribute valuable insights to the evolving understanding of rehabilitation strategies for individuals recovering from lingering impacts of COVID-19.

## Declaration

We used assistance from artificial intelligence (i.e. language model, ChatGPT 3.5) in the correction and improvement of our scientific paper [100].

## Name and location of the institution where the review was performed

Fattouma Bourguiba hospital Monastir, Tunisia.

## Supplementary Material

Effects of a cardiopulmonary rehabilitation programme on submaximal exercise in Tunisian patients with long-COVID19:A randomized clinical trial
