# Supplementary material for: Effects of a cardiopulmonary rehabilitation programme on submaximal exercise in Tunisian patients with long-COVID19: A randomized clinical trial
Source: Biol Sport. 2024 Apr 25;41(4):197–217. doi: 10.5114/biolsport.2024.139072 (PMC11474993; doi:10.5114/biolsport.2024.139072)
Supplement: Effects of a cardiopulmonary rehabilitation programme on submaximal exercise in Tunisian patients with long-COVID19:A randomized clinical trial [file JBS-41-54004-s1.pdf]

APPENDIX

TO PAPER: Kaddoussi R, Rejeb H, Kalai A et al. Effects of a cardiopulmonary rehabilitation programme on submaximal exercise in Tunisian patients with long-COVID19: A randomized clinical trial. Biol Sport. 2024;41(4):197–217. (link)

INTRODUCTION

As of December 30, 2023, 26 RCTs related to the effects of CPRPs on LC19Ps have been published in *PubMed* [*ie*; research using the two keywords (pulmonary rehabilitation) AND (COVID-19)] [1–26], but only 11 have exclusively reported 6MWT and/or pulmonary function data, among others [1–11].

MATERIALS AND METHODS

Second phase (2 weeks): Pre-CPRP' evaluations

The mMRC scale is a self-rating scale that measures the disability caused by breathlessness in daily activities [27]. This scale ranges from level 0 to level 4, where '0' is no breathlessness, except on strenuous exercise; and '4' is too breathless to leave the house, or breathless when dressing or undressing [27].

DISCUSSION

Rationale for choosing the 6MWD data as main outcome

The assessment of exercise tolerance is typically determined by measuring the maximum oxygen consumption in a cardiorespiratory test conducted under closely monitored conditions, with both a physician and a technician present [28]. Nevertheless, conducting this cardiorespiratory test requires sophisticated and costly equipment, along with personnel possessing advanced skills for its operation [28]. As a result, the repeated utilization of this test imposes a significant financial burden and is not feasible on a broad scale [28]. These constraints have prompted the adoption of simpler assessments, like the 6MWT [29–31]. The 6MWT comes with various benefits, such as increased safety, straightforward administration, improved patient tolerance, and a closer correlation with everyday activities [29–31]. Moreover, it is cost-effective and can be easily implemented on a large scale [29–31]. Extraordinarily, the 6MWT can be successfully completed by many elderly, frail, and severely restricted patients who are unable to undergo conventional maximal cycle ergometer or treadmill exercise tests [32]. This test entails measuring the distance an individual can walk on a flat surface within a span of 6 minutes [29–31]. Its increasing popularity can be attributed to its simplicity and the fact that it does not require complex equipment, rendering it suitable even for severely debilitated patients [29–31]. Over the last two decades (i.e., 2004–2024), the 6MWT has been extensively used in preoperative and postoperative evaluations [29–31, 33–37] and in assessing functional exercise performance across diverse populations including those with chronic pulmonary, cardiac, renal, metabolical, and neuromuscular conditions, scleroderma, infection with human immunodeficient virus, and ageing [29–77]. Furthermore, it has been demonstrated that the 6MWT independently predicts the risk of mortality among individuals with chronic illnesses [29, 30, 32, 44, 67, 68]. Its ability to independently predict mortality risk makes it a valuable tool for patient assessment and risk stratification [29–32].

Discussion of the methodology

The discrepancies noted between our results and these of some related RCTs (Tables 1S to 6S, Appendix) could be explained by at least 12 points related to differences in: *(i)* Recruitment methods, *(ii)* Applied questionnaires and tests, *(iii)* Blinding technique and randomization method, *(iv)* Primary and secondary outcomes, *(v)* Time interval between the onset of COVID-19 and the initiation of the CPRP, *(vi)* Applied inclusion, non-inclusion and exclusion criteria, *(vii)* Sample size calculation, *(viii)* Characteristics of LC19Ps and controls, *(ix)* Methodological aspects of the 6MWT, *(x)* Collected data during the 6MWT, *(xi)* Methodological aspects of the CPRP, and *(xii)* Statistical approaches. Discussion related to points *(i)* to *(vi)* is exposed in the following sentences.

Recruitment methods

As previously done in three related RCTs [4–6], our patients were recruited from the consultants of a department of pulmonology outpatient. In some similar RCTs (Table 2S), various recruitment strategies were utilized such as patients contacted by telephone [1, 2], or patients who were transferred directly from intensive care unit after a severe course of COVID-19 [11].

Applied questionnaires and tests

As previously done in four related RCTs [2, 6, 9, 10], we have applied a general questionnaire involving demographic, clinical and COVID-19 data, and we have evaluated dyspnea via the Borg [7, 8, 10] and the mMRC [1, 4–8, 10] scales. In related RCTs, various questionnaires were used (Table 1S) [*eg*; activities of daily living questionnaire [3], self-rating depression scale [3], self-rating anxiety scale [3], hospital anxiety and depression [8, 9], short form health survey-36 [3, 8], short form health survey-12 [1], Saint George's respiratory questionnaire [4, 8], VQ11 [5], multidimensional fatigue inventory [5], symptoms score [7], and fatigue severity scale [6]].

Similar to seven related RCTs [1–5, 8, 10], we have practiced 6MWTs and spirometric tests. In related studies (Table 1S), in addition to the 6MWT, further tests were utilized such as respiratory muscle strength [2], maximum static inspiratory pressure [6], 30-second sit-to-stand test, static squat test [1], five-time sit to stand [2], 1-minute sit to stand [5],timed up and go test [2], handgrip strength [2, 11], hemodynamic and vascular data [2], arterial blood pressure [6], Berg scale [11], Tinetti scale [11], isokinetic muscle force test [11], Barthel index [11], functional independence measure [11], and serum lactate lev\el [6].

Blinding technique and randomization method

As done in some related RCTs (Table 1S), we have opted for

randomization [1, 4–10] and blinding [1, 2, 6]. Problematic randomization and the absence of blinding of patients and training performers, may undermine the validity and reliability of individual study results or data synthesis [78].

Primary and secondary outcomes

As done in five related RCTs [1, 4, 5, 8, 10] (Table 1S), our main outcome was the 6MWD. In the remaining RCTs (Table 1S), the primary outcomes were spirometric data [3, 4, 10], maximum static inspiratory pressure [6], dyspnea [7, 10], and fatigue and stress levels [10]. As done in some related RCTs [1, 4–6, 8] (Table 1S), our secondary outcomes were dyspnea and spirometric data.

Time interval between the onset of COVID-19 and the initiation of the CPRP

In our study, the time interval between the onset of COVID-19 and the initiation of the CPRP was at least 3-months. In related RCTs, these intervals were various (*eg*; period of at least four weeks [6, 8], three months [11] or six months [3, 11] following the onset of other acute diseases; mean of a 141 days [5]). These specific timeframes were defined to characterize the stage of COVID-19 recovery at the start of the CPRP in each respective RCT.

Applied inclusion, non-inclusion, and exclusion criteria

The inclusion, non-inclusion, and exclusion criteria applied in our RCT are similar to these of similar related studies (Table 2S). First, in line with some related RCTs [1, 8] (Table 2S), only LC19Ps with persistent dyspnea were included. Second, as done in some other studies (Table 2S), we have not included LC19Ps with some chronic conditions [1–11], and having 6MWT [2, 5] or spirometry [5] contra-indications. It appears that our RCT is the first to exclude active smokers (Table 2S). Third, as done in similar related studies [1, 2] (Table 2S), we have excluded files of patients who missed any session of the CPRP or who did not attend the final evaluation.

RESULTS

Effects on 6MWD

Our findings and these of the 11 other RCTs (Table 6S) concerning the effect of CPRP on 6MWD are in line with those reported by some previous seven SRs [78–84] who confirmed the effectiveness of CPRPs in enhancing various health outcomes, including physical health, in LC19Ps. The following sentences will discuss the findings of the aforementioned six SRs [78–84].

First, a SR including eight studies [79] have reported that CPRP: *i)* Produced significant improvement in 6MWD in COVID-19 patients as compared to the CG with a minimal difference (MD) of 66 m; *ii)* Was effective in improving 6MWD in both acute (*ie*; MD of 83 m) and chronic (*ie*; MD of 44.16 m) COVID-19 patients as compared to CG [79]; *iii)* Improves both mild (*ie*; MD of 72 m) and moderate/severe (*ie*; MD of 50 m) patients [79]; *iv)* Was effective in improving 6MWD in both face-to-face (*ie*; MD of 41 m) and telerehabilitation (*ie*; MD of 76 m) programs; and *vi)* Induces significant benefits, as compared to no intervention, from even two weeks (*ie*; MD of 78 m).

Second, a SR including six studies [80] have noted that CPRP improved 6MWD (*ie*; standardized mean difference (SMD) of 0.83) compared to the standard treatment CG.

Third, a SR including three studies [78] reported results from 6MWD to demonstrate improvement made on exercise capacity, however, large variation was observed across baseline data on 6WMT. The authors noted that the pooled estimate of effect of CPRP on 6MWD (*ie*; MD of 50 m) was in favor of IG, and superior to the recommend MCID [78].

Fourth, a SR including 11 studies [81] reported that 6MWT was used in five ones. On the one hand, qualitatively all the included studies reported increases in physical performance post-CPRP at the end of the treatment in LC19Ps. When CRPP is compared with the usual care or educational approaches, statistically significant differences favoring the IG in terms of physical function were noted [81]. However, when compared to general exercise, there were no differences between the groups in terms of physical function improvements [81]. When manual therapy was added to a CPRP, compared to CPRP alone, no significant differences were found between the groups [81]. On the other hand, concerning the quantitative analysis, the pooling of five RCTs [1, 85–88] identified no significant effects on physical function improvement, with higher pre-post intervention changes in the IG compared to the CG. Among the observational studies, five ones were pooled [89–93], and no significant effects were observed in the pre-post intervention change in physical performance (*ie*; mean of 94 m). The 6MWD pooled data from the observational studies [89–93] showed significant increase scores post-CPRP (*ie*; mean of 0.537 m).

Fifth, a SR including five studies [82], where only two evaluated the 6MWT [3, 94], reported significant differences pre- and post- the CPRP.

Sixth, a SR and meta-analysis on telerehabilitation in LC19Ps [83], which included four RCTs [1, 85, 87, 95] reported improvements in 6MWT following breathing exercises and non-specific strength and resistance exercises lasting from one to six weeks.

Finally, a SR [84] including six RCTs [1–4, 6, 7] provided compelling evidence supporting the effectiveness of CPRPs in improving submaximal exercise performance in LC19Ps.

Effects on dyspnea

Some of previous SRs [79–82, 96] have reported findings related to the effects of CPRP on dyspnea of LC19Ps. The following sentences will discuss the findings of the aforementioned five SRs [79–82, 96].

First, a SR including eight studies [79] have reported that CPRP: *i)* Resulted in a significant reduction in dyspnea in the IG as

compared to the CG (*ie*; SMD of -2.11); *ii)* Such as face-to-face and telerehabilitation programs induce benefits in both mild and moderate/severe patients; *iii)* Is superior to no intervention in reducing dyspnea in patients with both acute (*ie*; SMD of -2.42) and chronic (*ie*; MD of -0.88) COVID-19; and *iv)* Induces benefits, as compared to no intervention, from even two weeks (*ie*; MD of -5.02). The observed decrease in dyspnea perception during CPRP might be due to physiological adaption to exercise training [97].

Second, a SR including six studies [80] have identified that CPRP improved dyspnea (*ie*; SMD of 0.55) in the IG compared to CG.

Third, a SR including five studies [96] highlighted that the two ones who assessed the effects of CPRP on dyspnea in LC19Ps, have detected an improvement in dyspnea and perceived exertion in favor of the IG at the end of the intervention (*ie*; six weeks).

Fourth, a SR including 11 studies [81] have used two analysis approaches. From a qualitative point of view, the 11 studies reported improvements in dyspnea levels post-CPRP in LC19Ps at the end of their treatments [81]. When CPRP was compared to the usual care, with no controls or educational instructions, statistically significant differences were found between the groups, favoring intervention in terms of dyspnea improvement [81]. However, when compared to general exercise, no significant differences were found [81]. The addition of myofascial release therapy to a CPRP, compared to CPRP alone, resulted in statistical differences that favored the IG [81]. Regarding the quantitative analysis, contrary results were found [81]. Pooling of the included RCTs (n = 5) did not result in significant effects on dyspnea improvement. In case of the observational studies (n = 5), the same scenario occurs, and no significant effects were observed in the pre-post intervention changes [81].

Fifth, a SR who included five studies [82], noted that two prospective intervention studies [3, 94] have evaluated dyspnea, showing highly significant results between pre- and post- CPRP.

Table 1S. Methodological characteristics of some relative randomized controlled trials (RCT).

1st author	Liu [3]	Li [1]	do Amaral [2]	Nagy [6]	Okan [4]
Yr-publication	.2020	.2022	.2022	.2022	.2022
Country	.China	.China	.Brazil	.Egypt	.Turkey
Aim(s) related to this paper proposes: To	.Investigate the effects of 6-W respiratory rehabilitation training on respiratory function, QoL, mobility and psychological function in elderly C19P	.Investigate superiority of a TRT for COVID-19 over no rehabilitation with regard to exercise capacity, lower LMS, pulmonary function, QoL and dyspnea	.Test the hypothesis that tele-supervised home-based exercise training is an effective strategy for improving cardiovascular, respiratory, and functional capacity parameters in individuals that were hospitalized due to COVID-19	.Determine whether the addition of manual diaphragm release to an IMT program is more effective than IMT alone in reducing blood pressure, dyspnea, fatigue, and aerobic performance capacity in males with PC19S	.Evaluate the effectiveness of breathing exercises given by telemedicine in PC19S dyspneic individuals
Study design	.Open label RCT<br>.Observational<br>.Prospective<br>.Quasi-experimental	.Multicenter<br>.Parallel-group RCT	.RCT<br>.Single-center<br>.Single-blinded	.Single-blinded RCT	.Pretest-posttest<br>.RCT parallel group study
Study period	.NR	.2020<br>.2 evaluations: 6 Ws (post-treatment) and 24 Ws (follow-up)	.NR	.2021 to 2022	.2020 to 2021
Sample size	.Calculated	.Calculated	.Not calculated	.Calculated	.Calculated
Target C19P	.Patients discharged from the hospital with satisfying results	.Patients recovering from COVID-19 and discharged from hospitals	.Patients that were hospitalized at the hospital	.Males with PC19S	.Patients who: *Received treatment for COVID-19<br>*Completed 2 months after treatment<br>*Presented to the chest diseases outpatient clinic with dyspnea

Table 1S. Continue

1st author	Liu [3]	Li [1]	do Amaral [2]	Nagy [6]	Okan [4]
Applied questionnaires	.ADL<br>.SF-36<br>.SDS<br>.SAS	.SF-12<br>.mMRC	.Anamnesis (demographic and health characteristics, persistent symptoms? medications used)	.Personal information form<br>.mMRC<br>.Fatigue severity scale	.SGRQ<br>.mMRC
Applied tests	.Spirometry<br>.6MWT	.6MWT<br>.Static squat test<br>.Spirometry	.Anthropometric data<br>.Hemodynamic data<br>.Vascular data<br>.Ventilatory data (spirometry, respiratory muscle strength)<br>.Functional data (HGS, FTSTS, TUG]<br>.6MWT	.6MWT<br>.Arterial BP<br>.Serum lactate level<br>.Maximum static inspiratory pressure	.Spirometry<br>.6MWT
Blinding technique	.NR	.Evaluators (rehabilitation doctor and therapist)	.Evaluators<br>.Observers	.Patients<br>.Outcome analyzer	.NR
Randomization method	.NR	.Permutated allocation sequences for 1:1 block randomization (block size 10–14)	.NR	.Patients were randomized 1:1 by an independent statistician<br>.Each patient picked an opaque sealed envelope, numbered sequentially by a researcher who was not involved in the study	.An online randomization site<br>.Sequentially numbered, opaque, sealed envelopes containing the assignment<br>.Opaque envelopes in a locked cupboard were opened sequentially by an external independent person before the participants
Primary outcome	.Spirometric data	.6MWT data	.NR	.Maximum static inspiratory pressure	.Spirometry<br>.6MWT
Secondary outcomes	.6MWD<br>.ADL<br>.QoL<br>.Anxiety score<br>.Depression score	.LMS<br>.Spirometric data<br>.QoL,<br>.Dyspnea	.NR	.mMRC<br>.6MWT<br>.Arterial BP<br>.Fatigue severity scale<br>.Serum lactate level	.SGRQ<br>.mMRC

1st author	Pang [7]	Vallier [5]	Şahın [8]	Rutkowski [9]	Rutkowski [10]	Trzmiel [11]
Yr-publication	.2022	.2023	.2023	.2022	.2023	.2023
Country	.China	.France	.Turkey	.Poland	.Poland	.Poland
Aim(s) related to this paper proposes: To	.Evaluate the effectiveness and safety of QJYQ on PC19S	.Investigate whether home-based rehabilitation would have similar effects compared to inpatient rehabilitation on physical and respiratory variables in PC19Ps.	.Compare the effects of a home-based pulmonary rehabilitation program with and without telecoaching on health-related outcomes in C19Ps' survivors	.Investigate and compare an innovative in-hospital pulmonary rehabilitation programs augmented with training elements performed in virtual reality	.Evaluate if a 3-W pulmonary rehabilitation program will improve the pulmonary function, exercise capacity and the stress level of individuals with PASC19	.Compare the effectiveness of traditional neurological rehabilitation and neurological rehabilitation combined with a rehabilitation robot for patients with PC19FS
Study design	.RCT<br>.Open-label	.RCT	.RCT	.RCT		.RCT<br>.Open access
Study period	.NR	.2021	.2021	.NR		.2022
Sample size	.Not calculated	.Calculated	.Calculated	.Calculated		.Not Calculated

Table 1S. Continue

1st author	Pang [7]	Vallier [5]	Şahın [8]	Rutkowski [9]	Rutkowski [10]	Trzmiel [11]
Target C19P	.Mild, ordinary, or severe C19P	.Patients with at least one physical or respiratory sequela and discharged from hospitals	.Patients who completed medical treatment	.Patients previously affected by COVID-19		.Participants who were transferred directly from intensive care units due to post-viral fatigue after experiencing a severe course of COVID-19
Applied questionnaires	.mMRC<br>.BS<br>.Symptoms score	.mMRC<br>.MFI<br>.VQ11	.mMRC<br>.HAD<br>.SGRQ<br>.SF-36<br>.Modified BS	.Self-administered sociodemographic questionnaire<br>.HAD<br>.WHOQOL-BREF	.Self-administered sociodemographic questionnaire<br>.mMRC<br>.BS<br>.PSS-10	.NR
Applied tests	.6MWT	.Spirometry<br>.6MWT<br>.1-minute sit to stand<br>.Squat jump	.6MWT<br>.Spirometry	.6MWT	.6MWT<br>.Spirometry	.6MWT<br>.HGS<br>.Berg scale<br>.Tinetti scale<br>.Isokinetic muscle force test<br>.Barthel index<br>.Functional independence measure
Blinding technique	.NR	.NR	.NR	.NR		.NR
Randomization method	.Randomization in a 1:1 ratio	.Web-based randomization	.Randomization program	.Web Randomizer (1:1 ratio)		.NR
Primary outcome	.mMRC<br>.BS	.6MWT data	.6MWT data	.NR	.Spirometric data<br>.6MWT<br>.Fatigue level<br>.Dyspnea level<br>.Stress level	.NR
Secondary outcomes	.6MWT<br>.Symptoms score	.Spirometric data<br>.1-minute sit to stand<br>.Force and power of the lower limb<br>.MFI scores<br>.VQ11 score	.LMS<br>.Spirometric data<br>.SGRQ<br>.SF-36<br>.Dyspnea<br>.Muscle strength	.NR		.NR

ADL: Activities of daily living. BP: Blood pressure. BS: Borg scale. C19P: COVID-19 patients. FTSTS: Five-time sit to stand. HAD: Hospital anxiety and depression. HGS: Handgrip strength. LMS: Limb muscle strength. MFI: Multidimensional fatigue inventory. mMRC: Modified medical research council. NR: Not reported. PASC19: post-acute sequelae of COVID-19. PC19FS: post-COVID-19 fatigue syndrome. PC19P: post COVID-19 patient. PC19S: Post-COVID-19 syndrome. PSS-10: Perceived stress scale. QJYQ: Qingjin yiqi granules. QoL: Quality of life. SAS: Self-rating anxiety scale. SDS: Self-rating depression scale. SF-12: Short form health survey-12. SF-36: Short form-36. SGRQ: St. George's respiratory questionnaire. TRT: Telerehabilitation tools. TUG: Timed up and go test. VQ11: QoL questionnaire. W: Week. WHOQOL-BREF: Short version of the world health organization QoL questionnaire. Yr: Year. 6MWD: 6-min walk distance. 6MWT: 6-minute walk test.

Table 2S. Recruitment methods, inclusion, non-inclusion and exclusion criteria applied in some relative randomized controlled trials (RCT).

1st author	Liu [3]	Li [1]	do Amaral [2]	Nagy [6]	Okan [4]
Recruitment method	.Patients who consulted hospitals designated to admit C19P	.Telephone contact	.Telephone call	.Patients from chest disease department outpatient clinic	.C19P who presented to the chest diseases outpatient clinic with dyspnea
Inclusion criteria	.Diagnosis of COVID-19<br>.Age $\geq$ 65 yrs<br>.$\geq$ 6 months after the onset of other acute diseases<br>.MMSE score > 21<br>.No COPD<br>.No respiratory disease<br>.FEV_1 ≥70%	.Age: 18–75 yrs,<br>.Discharged from hospitals after inpatient treatment for COVID-19<br>.mMRC: 2–3	.Diagnosis of COVID-19<br>.Age: $\geq$ 18 yrs	.Males<br>.Age: 30–45 yrs<br>.BMI: 25–29.9 kg/m^2<br>.Low-moderate PA (IPAQ)<br>.Mild-Moderate lung fibrosis (high-resolution chest CT)<br>.AHT at stage II (160–179/100–109 mmHg)<br>.Stable and non-hospitalized after COVID-19<br>.At least 4 Ws since the first positive COVID-19 swab at the time of screening	.Age > 18<br>.Access to the appropriate communication device or internet for telemedicine implementation<br>.No visual-impairment<br>.No disease that could prevent mobilization
Non-inclusion criteria	.Moderate-severe heart disease (grade III or IV, NYHA)<br>.Severe ischemic<br>.Hemorrhagic stroke<br>.Neurodegenerative diseases	.mMRC: 4–5<br>.Resting HR > 100 bpm<br>.Uncontrolled ATH<br>.Uncontrolled chronic disease (eg, DM with RBG > 16.7 mmol/L, HbA_1C > 7.0%)<br>.Cerebrovascular disease within 6 months<br>.Intra-articular drug injection or surgical treatment of lower extremities within 6 months<br>.Medication affecting cardiopulmonary function such as bronchodilators or β-blockers<br>.Unable to walk independently with assistive device<br>.Unable or unwilling to collaborate with assessments<br>. Enrolment or participation in other trials within past 3 months<br>.History of severe cognitive or mental disorder or substance abuse<br>.Enrolment in other rehabilitation program	.Contraindications for PA (*ie*; recent MI, unstable angina or arrhythmias or other uncontrolled heart disease)<br>.Decompensation<br>.Metabolic, pulmonary, hepatic or renal diseases<br>.Pregnant or lactating females	.Cardiac disease<br>.Chronic respiratory disease<br>.Active infection<br>.Severe endocrine/ metabolic diseases<br>.Cognitive impairment<br>.Disabilities that interfered with the intervention<br>.Reasons that made the participants ineligible for participation	.FEV_1 < 50%<br>.$FEF_{25–75}$ < 50%<br>.Resting SpO_2 < 85%<br>.Moderate-advanced heart failure<br>.Disease that could limit mobility<br>.Advanced stage liver and kidney failure<br>.MI within the last 4 months<br>.History of unstable angina and active infectious disease

Table 2S. Continue

1st author	Liu [3]	Li [1]	do Amaral [2]	Nagy [6]	Okan [4]
Exclusion criteria	During the 6MWT:<br>.Worsening of dyspnea,<br>.SpO_2 decrease ≤ 85%<br>.HR increase ≥ 85% predicted maximum HR	.Missed assessments<br>.No compliance	.No participation in follow-up evaluation	.Red flag indicators (chest pain, critical drop in SpO_2)<br>.Musculoskeletal or neurological limitations<br>.Unconscious patients<br>.Comorbidities<br>.Participation in a clinical study or other research in the previous 30 days	.NR

1st author	Pang [7]	Vallier [5]	Şahın [8]	Rutkowski [9, 10]	Trzmiel [11]
Recruitment method	.Patients who were hospitalized at 2 hospitals during the treatment period	.PC19P from the respiratory disease unit	.Patients who were hospitalized at the ICU or ward for more than 10 days	.Patients enrolled in post-COVID-19 rehabilitation at a hospital	.Patients who were transferred directly from ICU after a severe course of COVID-19
Inclusion criteria	.2 consecutive negative results of RT-PCR tests with at least a 1-day interval between tests<br>.Mild-severe C19P<br>.Substantial enhancements of acute exudative lesions on chest radiography<br>.Discharge criteria<br>.Normal temperature > 3 days<br>.Significant improvements in respiratory symptoms	.Age: > 18 yrs<br>.Recent COVID-19 disease proved by RT-PCR test<br>.At least 1 physical and/or respiratory sequela post COVID-19<br>.Internet access with videoconference appliances in working order	.Diagnosis of COVID-19<br>.Stay in the ICU or ward for more than 10 days with or without the need for IMV<br>.Receipt of non-IMV<br>.Receipt of high-flow O_2 therapy<br>.Complete of medical treatment<br>.Receipt of outpatient pharmacological treatment before hospitalization<br>.Dyspnea for the 1st time ever because of the disease<br>.Dyspnea continued despite treatment	.Ag: 40–80 yrs<br>.Diagnosis of COVID-19	.Diagnosis of COVID-19<br>.Transfer from the ICU<br>.Symptoms present for more than 6 months<br>.At least 3 main criteria (*ie*; deterioration of the ability to perform tasks carried out before the disease; severe fatigue not improved by rest; discomfort after physical activity; non-restorative sleep) and 1 additional criterion (*ie*; cognitive impairment, orthostatic intolerance)
Non-inclusion criteria	.Chronic lung diseases (*eg*; COPD, ILD)<br>.History of pulmonary surgical intervention<br>.Severe clinical condition (*eg*; heart failure, stroke)<br>.Severe chronic disease (*eg*; liver cirrhosis, malignant tumors)<br>.Allergic constitution<br>.Allergic to QJYQ<br>.Pregnant or breast feeding females	.Cardiovascular contraindications to exercise<br>.Respiratory instability<br>.Neuromuscular, articular, or psychiatric disease preventing physical exercise	.Past the post-acute phase<br>.Orthopedic problems<br>.Receipt of treatment for active cancer<br>.Cardiac and thromboembolic complications	.No consent<br>.Active pneumonia<br>.Heart disease (stable or unstable)<br>.Uncontrolled ART<br>.Insulin-dependent DM<br>.Inability to exercise independently<br>.Musculoskeletal/ neurological conditions that would prevent the completion of the course<br>.Lung cancer<br>.Cognitive impairment or MMSE < 24	.Active medical condition (infections; tumors; rheumatological, metabolic, endocrine, autoimmunological, cardiovascular diseases)<br>.Bipolar disorder<br>.Dementia<br>.Nutritional disorders<br>.Addiction to alcohol or psychoactive substances<br>.Severe obesity<br>.Overtraining
Exclusion criteria	.Other herbal medicines	.NR	.NR	.NR	.NR

AHT: Arterial hypertension.BMI: Body mass index. C19P: COVID-19 patients. COPD: Chronic obstructive pulmonary disease. CT: Computed tomography. DM: Diabetes mellitus. $FEF_{25–75\%}$: Forced mid-expiratory flow. FEV_1: Forced expiratory volume in 1 second. HbA_1C: Hemoglobin A_1C. HR: Heart rate. ICU: intensive care unit.ILD: Interstitial lung disease. IMV: invasive mechanical ventilation. IPAQ: International physical activity questionnaire. MI: Myocardial infarction. mMRC: Modified medical research council. MMSE: Mini-mental state examination. NYHA: New York heart association. PA: Physical activity. PC19P: post-COVID-19 patient. PCR: Polymerase chain reaction. QJYQ: Qingjin yiqi granules. RBG: Random blood glucose. RT-PCR: Reverse transcriptase-polymerase chain reaction. SpO_2: Oxy-hemoglobin saturation. W: Week. Yr: year. 6MWT: 6-minute walk test.

Table 3S. Characteristics of participants included in some relative randomized controlled trials (RCT).

1st author	Groups	Liu [3]	Li [1]	do Amaral [2]	Nagy [6]	Okan [4]
Sample size (M/F)	Cases	.36 (24/12)	.59 (27/32)	.12 (7/5)	.26 (26/0)	.26 (15/11)
	Controls	.36 (25/11)	.60 (26/34)	.20 (8/12)	.26 (26/0)	.26 (12/14)
Age (year)	Cases	.69 ± 8[a]	.49 ± 11[a]	.52 ± 10[a]	.40 ± 3[a]	.49 ± 11[a]
	Controls	.68 ± 8[a]	.52 ± 11[a]	.53 ± 12[a]	.40 ± 3[a]	.52 ± 15[a]
BMI (kg/m²)	Cases	.23.1 ± 3.5[a]	.NR	.32.6 ± 7.4[a]	.27.4 ± 1.5[a]	.31.1 ± 4.2[a]
	Controls	.22.9 ± 3.9[a]	.NR	.32.4 ± 7.8[a]	27.6 ± 1.2[a]	31.3 ± 4.4[a]
Corpulence status	Cases	.NR	.Obesity:15.3[b]	.Obesity: 67[b]	.Overweight:100[b]	.NR
	Controls	.NR	.Obesity:13.33[b]	.Obesity: 65[b]	.Overweight:100[b]	.NR
COVID-19 diagnosis method		.NR	.NR	.RT-PCR test	.NR	.NR
COVID-19 severity	Cases	.NR	.Severe: 37[b]	.NR	.NR	.NR
	Controls	.NR	.Severe: 27[b]	.NR	.NR	.NR
Extent of lung CT lesion	Cases	.Multimodal lesion:69[b]<br>.Unilobulaire lesion:31[b]<br>.Pleural effusion:11[b]	.NR	.NR	.Mild fibrosis:33[b]<br>.Moderate fibrosis:67[b]	.NR
	Controls	.Multimodal lesion:64[b]<br>.Unilobulaire lesion:36[b]<br>.Pleural effusion:8[b]	.NR	.NR	.Mild fibrosis:40[b]<br>.Moderate fibrosis:60[b]	.NR
Comorbidities	Cases	.AHT:27.8[b]<br>.DM:25.0[b]<br>.Osteoporosis:22.2[b]	.No comorbid:42.4[b]<br>.Heart disease:3.4[b]<br>.AHT:13.6[b]<br>.DM:13.6[b]<br>.Lung diseases:6.8[b]<br>.Other:27.1[b]	.No comorbid:25[b]<br>.AHT:42[b]<br>.DM:33[b]<br>.Heart diseases:0[b]<br>.Dyslipidaemia:8[b]<br>.Lung diseases:8[b]<br>.Hypothyroidism:0[b]<br>.Other:25[b]	.NR	.Chronic disease:53.8[b]<br>.AHT:19.2 [b]<br>.DM:11.5 [b]<br>.CAD:11.5 [b]<br>.CRF:3.7 [b]<br>.Hypothyroidism:7.7 [b]
	Controls	.AHT:22.2[b]<br>.DM:25.0[b]<br>.Osteoporosis:16.7[b]	.No comorbid:35.0[b]<br>.Heart disease:11.7[b]<br>.AHT:30[b]<br>.DM: 15[b]<br>.Lung diseases:5[b]<br>.Other:20[b]	.No comorbid:15[b]<br>.AHT:55[b]<br>.DM:5[b]<br>.Heart diseases:10[b]<br>.Dyslipidaemia:10[b]<br>.Lung diseases:10[b]<br>.Hypothyroidism:5[b]<br>.Other:20[b]	.NR	.Chronic disease:50 [b]<br>.AHT:19.2 [b]<br>.DM:15.4 [b]<br>.CAD:11.5[b]<br>.CRF:0[b]<br>.Hypothyroidism:3.8[b]
Smoking history	Cases	.NR	.15.3[b]	.25[b]	.63.4[b]	.23.1[b]
	Controls	.NR	.10.0[b]	.25[b]	.53.3[b]	.15.4[b]

1st author	Groups	Pang [7]	Vallier [5]	Şahın [8]	Rutkowski [9, 10]	Trzmiel [11]
Sample size (M/F)	Cases	.194 (72/122)	.8 (7/1)	.21 (13/8)	.18 (7/11)	.42 (NR)
	Controls	.194 (77/117)	.9 (5/4)	.21 (15/6)	.14 (5/9)	.39 (NR)
Age (year)	Cases	.48 ± 1[c]	.57 ± 19[a]	.58 ± 8[a]	.59 ± 4[a]	.67 ± 8[a]
	Controls	.45 ± 1[c]	.53 ± 14[a]	.64 ± 8[a]	.56 ± 7[a]	.65 ± 12 [a]
BMI (kg/m²)	Cases	.NR	.33.0 ± 5.9[a]	.30.0 ± 6.4[a]	.NR	.28.2 ± 3.5[a]
	Controls	.NR	.27.9 ± 6.6[a]	. 28.7 ± .5[a]	.NR	.25.6 ± 3.9[a]
Corpulence status	Cases	.NR	.NR	.NR	.NR	.NR
	Controls	.NR	.NR	.NR	.NR	.NR
COVID-19 diagnosis method		.NR	.NR	.RT-PCR test	.NR	.NR
COVID-19 severity	Cases	.Mild/severe	.NR	.NR	.NR	.NR
	Controls	.Mild/severe	.NR	.NR	.NR	.NR

Table 3S. Continue

1st author	Groups	Pang [7]	Vallier [5]	Şahın [8]	Rutkowski [9, 10]	Trzmiel [11]
Extent of lung CT lesion	Cases	.NR	.NR	.Bilateral:74[b]<br>.Unilateral:42[b]<br>.Pleural effusion:5[b]<br>.Ground-glass opacity:74[b]<br>.Nodule:10[b]	.NR	.NR
	Controls	.NR	.NR	.Bilateral:42[b]<br>.Unilateral:58[b]<br>.Ground-glass opacity:84[b]	.NR	.NR
Comorbidities	Cases	.NR	.NR	.Comorbidity:74[b]	.NR	.NR
	Controls	.NR	.NR	.Comorbidity:68[b]	.NR	.NR
Smoking history	Cases	.NR	.NR	.Current smoker:5[b]<br>.Former smoker:42[b]<br>.Never smoker:53[b]	.NR	.NR
	Controls	.NR	.NR	.Current smoker:0[b]<br>.Former smoker:68[b]<br>.Never smoker:32[b]	.NR	.NR

Data were: [a]Mean ± SD; [b] %, [c]Mean ± SEM

ADL: Activities of daily living. AHT: Arterial hypertension. BMI: Body mass index. CAD: Coronary artery disease. COPD: Chronic obstructive pulmonary disease. CRF: Chronic heart failure. CT: Chest tomography. DM: Diabetes mellitus. F: Female. FEV_1: Forced expiratory volume in one second. M: Male. MMSE: Mini-mental state examination. NR: not reported. QoL: Quality of life. RT-PCR: Reverse transcriptase-polymerase chain reaction. SAS: Self-rating anxiety scale. SDS: Self-rating depression scale. SF-36: Short form-36. SpO_2: Oxy-hemoglobin saturation. Yr: Year.

Table 4S. Methodological aspects of the 6-minute walk test (6MWT) in some relative randomized controlled trials.

1st author		Liu [3]	Li [1]	do Amaral [2]	Nagy [6]	Okan [4]	Pang [7]	Vallier [5]	Şahın [8]	Rutkowski [9, 10]	Trzmiel [11]
6MWT practice											
Guidelines		.NR	.ERS/ATS 2014 [31]	.ERS/ATS 2014 [31]	.NR	.ATS 2002 [29]	.ATS 2002 [29]	.ATS 2002 [29]	.ATS 2002 [29]	.ERS/ATS 2014 [31]	.ATS 2002 [29]
Corridor length (m)		.NR	.30	.30	.35	.30	.NR	.NR	.30	.30	.30
Outdoor/Indoor		.NR	.Baseline: Outdoor .Follow-up: Indoor (hospital)	.NR	.Indoor	.NR	.NR	.Cases: Indoor .Controls: Outdoor	.NR	.NR	.Indoor
Collected data	6MWD	.Yes	.Yes	.Yes	.Yes	.Yes	.Yes	.Yes	.Yes	.Yes	.Yes
	HR	.Yes	.Monitored	.Yes	.NR	.NR	.NR	.NR	.NR	.NR	.NR
	Dyspnea	.Yes	.Yes	.No	.Yes	.Yes	.Yes	. Yes	.Yes	.Yes	.NR
	SpO_2	.Yes	.Monitored	.Yes	.NR	.NR	.NR	.NR	.NR	.NR	.NR
	SBP	.Yes	.Yes	.Yes	.NR	.NR	.NR	.NR	.NR	.NR	.NR
	DBP	.Yes	.Yes	.Yes	.NR	.NR	.NR	.NR	.NR	.NR	.NR
	Other	.RR	-	.Recovery BP, HR, SpO_2	-	-	-	-	-	.Fatigue	-
6MWD											
Expression mode	m	.Yes	.Yes	.Yes	.Yes	Yes	.Yes	.Yes	.Yes	.Yes	.Yes
	%Pred	.No	.No	.Yes	.No	.No	.No	.No	.No	.No	.No
Reference equations		.NR	.NR	.Yes	.NR	.Yes(98)	.NR	.NR	.NR	.NR	.NR
HR expression mode	bpm	.NR	.NR	.Yes	.NR	.NR	.NR	.NR	.NR	.NR	.NR
	%Max pred	.NR	.NR	.NR	-	.NR	.NR	.NR	.NR	.NR	.NR
Dyspnea scale		.BS	.mMRC	.NR	.mMRC	.mMRC	.mMRC .BS	.mMRC	.mMRC .modified BS	.BS	.No
Other remarks		-	.Patients allowed to use walking aids	.Partial oxygen desaturation during the exercise phase: reduction equal or greater than 4% in SpO_2	-	-	-	-	-	.Patient allowed to move independently and rest if necessary	-
6MWT data comparison											
Significant statistical approach		.Yes	.Yes	.Yes	.Yes	.Yes	.Yes	.Yes	.Yes	.Yes	.Yes
Significant clinical approach		.No	.Yes (MID of 50 m for the 6MWD)	.No	.No	.No	.No	.Sample size was determined to the primary outcome (6MWD) with a non-inferiority limit equal to 50 m	.No	.Yes (effect size calculated)	.No

ATS: American thoracic society. BP: Blood pressure. BS: Borg scale. DBP: Diastolic blood pressure. ERS: European respiratory society. HR: Heart rate. MID: Minimal important difference. mMRC: Modified medical research council. NR: Not-reported. RR: Respiratory rate. SBP: Systolic blood pressure. SpO_2: Oxy-hemoglobin saturation. %Max pred: Percentage of predicted maximal heart rate. %pred: Percentage of predicted value. 6MWD: 6-min walk distance.

Table 5S. Methodological aspects of the cardiopulmonary rehabilitation programs (CPRP) in some relative randomized controlled trials.

1st author	Liu [3]	Li [1]	do Amaral [2]	Nagy [6]	Okan [4]	Pang [7]
Location	.Hospital<br>.Home	.Home TR	.Tele-supervised (home)	.Hospital	.Hospital<br>.Home telemedicine	.Hospital
Number of session/W	.2	.3–4	.Resistance training: 3<br>.Aerobic training: 5	.Manual DR: 3<br>.IMT via POWERbreath: 3	At hospital: 21<br>(3 sessions/day<br>(10 exercises each)<br>Telemedicin: 1 breathing exercises<br>Walking training: 5	.NR
Number of Ws	.6	.6	.12	.6	.5	.2
Duration of a session (min)	.10	.NR	.Resistance training: NR<br>.Aerobic training:<br>.W1: 10–15<br>.W3 to 4: 20<br>.W5 to 12: 30	.Manual DR: session < 3<br>.IMT via POWERbreath: 4	.NR	.NR
Content of the CPRP for cases	.Respiratory muscle training<br>.Cough exercise<br>.Diaphragmatic training<br>.Stretching exercise<br>.Home exercise	.Breathing control<br>.Thoracic expansion<br>.Lower limb muscle strength exercises<br>.Aerobic exercise (intensity based on HR reserve, and ranged from 30–40% for tier 1 to 40–60% for tier 3)	.5 min warm-up: joint mobility and stretching exercises<br>.5 min of cool-down: stretching and relaxing exercises<br>.Resistance training: 9 multi- and single-joint exercises (bodyweight squat, push-up on the wall, bodyweight lunge, one-arm row, deadlift, side lateral raise or shoulder press, elbow flexion, calf raise, and abdominal crunch in chair) using body weight and/or rubber bands/plastic bottles (with water or sand) as resistance.<br>.W1: 1 set of 10–15 reps<br>.W2–3: 2 sets of 10–15 reps<br>.W4–6: 3 sets of 10–15 reps<br>.W7–12: 3 sets of 15–20 reps<br>.Rest: 1 min between sets<br>Intensity: 14–17 points of RPE<br>Aerobic training: walking and/or cycling<br>W1: 10–15 min<br>W3–4: 20 min<br>W5–12: 30 min<br>Intensity: 11–13 points of RPE	.Manual DR method: 1 physiotherapist applied this technique. 2 sets of 10 deep breaths, with a 1-min interval between them DR session: 3 min long<br>IMT via POWERbreath: .2 sets of 30 dynamic inspiratory efforts (2-min interval between sets) from an upright sitting position with a 4-min session length overall maximally, twice daily with a PImax workload of 60%	.Respiratory control<br>.Pursed lip breathing<br>.Diaphragmatic breathing exercises<br>.Mild-intensity walking program	Standard rehabilitation treatments<br>.Lip breathing training: 20 min each time, 3 times daily<br>.Abdominal breathing training: 7 breaths/min for 20 min, twice a day<br>.Respiratory rhythm training: 20 min each time, 3 times/day<br><br>Baduanjin exercise twice a day<br>QJYQ: dose of 10 g twice daily for 14 days

Table 5S. Continue

1st author	Liu [3]	Li [1]	do Amaral [2]	Nagy [6]	Okan [4]	Pang [7]
Content of the CPRP for controls	.Nothing	.Short educational instructions at baseline	.NR	.IMT	.Brochure explaining breathing control<br>.Pursed lip breathing<br>.Diaphragmatic breathing exercise.<br>First practice session: face-to-face in the hospital environment 20–30 min light-intensity Walk 5 times/W<br>.Aerobic exercise training: low-intensity ground-based walking training with a breathlessness or fatigue score of $<$ 3 (“moderate”) on the modified 0 to 10 BS	Standard rehabilitation treatments<br>.Lip breathing training: 20 min each time, 3 times daily<br>.Abdominal breathing training: 7 breaths/min for 20 min, twice a day<br>.Respiratory rhythm training: 20 min each time, 3 times/day
Guidelines	.NR	. [99]	.NR	.NR	. [100]	. [101]

1st author	Vallier [5]	Şahın [8]	Rutkowski [9]	Rutkowski [10]	Trzmiel [11]
Location	.Hospital<br>.Home	.Home	.Hospital		.Hospital
Number of session/W	. Walks: 4<br>.Endurance: 4<br>.Gymnastics/muscular strength: 3<br>.Sophrology:1	.Breathing exercises:5–7<br>.Strength training: 5–7<br>.Walking program:3–5	.5		.6
Number of Ws	.4	.8	.3		. 6
Duration of a session (min)	.Walks: 60<br>.Endurance: 40<br>.	.Walking program: 20–30	.NR		.Case group: 75/day, complemented by 45 min of exercises on the rehabilitation robot<br>.Control group: 120/day

Table 5S. Continue

1st author	Vallier [5]	Şahın [8]	Rutkowski [9]	Rutkowski [10]	Trzmiel [11]
Content of the CPRP for cases	.Endurance<br>.Ergocycle<br>20 min at 90–100% of the HR achieved at the end of the 6MWT<br>.10 min warm-up<br>.10 min cool-down	Breathing exercises:<br>.Techniques: diaphragmatic breathing, pursed-lip breathing, thoracic expansion exercises<br>.Frequency: 8–10 reps/set, 1–2 sets/day<br>Strength training:<br>.Intensity: perceived fatigue of ≤ 3 on the modified BS<br>.Frequency: 8–10 reps/set, 1–2 sets/day<br>.Progression: Weights were increased when perceived dyspnea and fatigue were ≤ 3 on the modified BS. Walking program:<br>.Intensity: Fatigue was targeted at ≤ 3 on the modified BS.<br>.Frequency: 20–30 min/day<br>.Progression: Walking speed and increased duration targeting a score of ≤ 3 on the modified BS for perceived dyspnea and fatigue	Ergocycle<br>(head-mounted display using "Virtual Park" software)<br>.Software brought the participants to a sunny island where they conducted a bike ride enhanced with realistic elements and sound effects simulating real-life situations<br>.Training HR limits vary by model (model A, 80% of the submaximal HR, model B, 70%, model C, 60%); in model D, the HR increases during the exercises by 20–30% in relation to the HR at rest<br>.Every procedure was performed once a day<br>.Relaxing provided<br>.Calmness and mood improvement<br>.Motivation and cognitive activation	Exercise training (cycle ergometer with a head-mounted display using "Virtual Park" software)<br>.Software brought the participants to a sunny island where they conducted a bike ride enhanced with realistic elements and sound effects simulating real-life situations<br>.Training HR limits vary by model (model A, 80% of the submaximal HR, model B, 70%, model C, 60%); in model D, the HR increases during the exercises by 20–30% in relation to the HR at rest<br>.Virtual Park software features an automatic system of changes in training HR: after reaching the target HR in the previous training session, the work rate on the last phase was increased by 5 Watts; if the HR was not reached, the final work rate was reduced by 5 Watts.<br>Breathing exercises<br>General fitness exercises<br>Resistance training<br>Relaxation<br>.Guided relaxation in a virtual setting using the goggles	Neurological rehabilitation<br>EMG rehabilitation robot<br>.Device helps patients with relatively low muscle strength perform active exercises.

Table 5S. Continue

1st author	Vallier [5]	Şahın [8]	Rutkowski [9]	Rutkowski [10]	Trzmiel [11]
Content of the CPRP for controls	.Endurance<br>.Walking for 20 min at 90–100% of the HR achieved at the end of the 6MWT<br>.10 min warm-up<br>.10 min cool-down	Breathing exercises:<br>.Techniques: diaphragmatic breathing, pursed-lip breathing, thoracic expansion exercises<br>.Frequency: 8–10 rep/set, 1–2 sets/day<br>Strength training:<br>.Intensity: perceived fatigue of ≤ 3 on the modified BS<br>.Frequency: 8–10 reps/set, 1–2 sets/day<br>.Progression: Weights were increased when perceived dyspnea and fatigue were ≤ 3 on the modified BS<br>Walking program:<br>.Intensity: Fatigue was targeted at ≤ 3 on the modified BS<br>.Frequency: 20–30 min/day<br>.Progression: Walking speed and increased duration targeting a score of ≤ 3 on the modified BS for perceived dyspnea and fatigue	Cycle ergometer without additional audio-visual stimuli<br>.Training HR limits vary by model (model A, 80% of the submaximal HR, model B, 70%, model C, 60%); in model D, the HR increases during the exercises by 20–30% in relation to the HR at rest<br>.Every procedure was performed once a day<br>Relaxing<br>.Schultz autogenic training<br>.		Neurological rehabilitation<br>.Neuromuscular re-education techniques<br>.Exercises for movement coordination and balance, with combined progressive endurance training.<br>.Maintain a standing position<br>.Active and resistance exercises<br>.Progressive endurance training: from 35% of maximum HR to 70% of maximum HR: 30 min/day
Guidelines	.NR	.NR	. [102]	. [103]	. [104]

BS: Borg scale. DR: Diaphragm release. HR: Heart rate. IMT: Inspiratory muscle training. min: Minutes. NR: Not-reported. PImax: Maximal inspiratory pressure. QJYQ: Qingjin Yiqi granules. Reps: Repetitions. RPE: Rating of perceived exertion. TR: Telerehabilitation. W: Week. 6MWT: 6-min walk test.

Table 6S. Results of the cardiopulmonary rehabilitation programs on 6-min walk distance (m) in some related randomized controlled trials.

1st author		Liu [3]	Li [1]	do Amaral [2]	Nagy [6]	Okan [4]	Pang [7]	Vallier [5]	Şahın [8]	Rutkows-ki [9, 10]	Trzmiel [11]
Cases	Pre	.163 ± 72[a]	.515 ± 83[a]	.522 ± 107[a]	.418 ± 19[a]	.424 ± 57[a]	.473 ± 7[d]	.447 ± 137[a]	.378 ± 29[d]	.502 ± 48[a]	.116 ± 112[a]
	Post	.212 ± 82[a]	.NR	.543 ± 115[a]	.474 ± 49[a*]	.478 ± 67[a*#]	.541 ± 6[d]	.542 ± 111[a]	.441 ± 26[d]	.558 ± 76[a]	.240 ± 124[a]
	Delta	.NR	.Post-treatment:80 ± 75[a] .Follow-up:85 ± 80[a]	-	.57 ± 48[a]	.54[cê]	-	.95[c]	.63 ± 15[d]	.57[c]	.NR
Controls	Pre	.156 ± 82[a]	.500 ± 93[a]	.472 ± 98[a]	.418 ± 19[a]	.414 ± 49[a]	.452 ± 7[d]	.542 ± 97[a]	.325 ± 29[d]	.512 ± 54[a]	.134 ± 121[a]
	Post	.157 ± 72[a]	.NR	.490 ± 76[a]	.435 ± 18[a]	.419 ± 54[a]	.538 ± 7[d]	.604 ± 88[a]	.382 ± 26[d]	.552 ± 49[a*]	.257 ± 124[a]
	Delta	.NR	.Post-treatment: 17 ± 64[a] .Follow-up: 15 ± 70[a]	-	.17 ± 10[a]	.5[c]	-	.72	.57 ± 15[d]	.39[c]	.NR
Comparisons	Group effect (Cases vs. Controls)	.Sig	.Sig	.NS	.Sig	.Sig	.NR	.NS	.NS	.NR	.NS
	Session effect (Pre vs. Post)	.Sig	.NR	.NS	.Sig	.Sig	.NR	.Sig	.Sig	.Sig	.Sig
	Group-session effect (comparison of Delta)	.NR	.NR	.NR	.Sig	.Sig	.NS	.NS	.NS	.NS	.NS
Effect size		.NR	.NR	.NR	.NR	.Large	NR	.NR	.NR	.Small	.NR

Data were: [a]Mean ± standard deviation; [b]%, [c]Mean, [d]Mean ± standard error of the mean.
Post: Post intervention. Pre: Pre intervention. Delta: Post minus pre. NR: Not-reported. NS: Non-significant. Sig: Significant.

REFERENCES

1. Li J, Xia W, Zhan C, Liu S, Yin Z, Wang J, et al. A telerehabilitation programme in post-discharge COVID-19 patients (TERECO): a randomised controlled trial. Thorax. 2022; 77(7):697–706. Epub 20210726. doi: 10.1136/thoraxjnl-2021-217382. PubMed PMID: 34312316; PubMed Central PMCID: PMC8318721.
2. Teixeira DOAV, Viana AA, Heubel AD, Linares SN, Martinelli B, Witzler PHC, et al. Cardiovascular, Respiratory, and Functional Effects of Home-Based Exercise Training after COVID-19 Hospitalization. Med Sci Sports Exerc. 2022; 54(11):1795–803. Epub 20220617. doi: 10.1249/MSS.0000000000002977. PubMed PMID: 35714077.
3. Liu K, Zhang W, Yang Y, Zhang J, Li Y, Chen Y. Respiratory rehabilitation in elderly patients with COVID-19: A randomized controlled study. Complement Ther Clin Pract. 2020; 39:101166. Epub 20200401. doi: 10.1016/j.ctcp.2020.101166. PubMed PMID: 32379637; PubMed Central PMCID: PMC7118596.
4. Okan F, Okan S, Duran Yucesoy F. Evaluating the Efficiency of Breathing Exercises via Telemedicine in Post-Covid-19 Patients: Randomized Controlled Study. Clinical nursing research. 2022; 31(5):771–81. Epub 20220429. doi: 10.1177/10547738221097241. PubMed PMID: 35485738.
5. Vallier JM, Simon C, Bronstein A, Dumont M, Jobic A, Paleiron N, et al. Randomized controlled trial of home-based vs. hospital-based pulmonary rehabilitation in post COVID-19 patients. Eur J Phys Rehabil Med. 2023; 59(1):103–10. Epub 20230126. doi: 10.23736/S1973-9087.22.07702-4. PubMed PMID: 36700245; PubMed Central PMCID: PMC10035444.
6. Nagy EN, Elimy DA, Ali AY, Ezzelregal HG, Elsayed MM. Influence of Manual Diaphragm Release Technique Combined with Inspiratory Muscle Training on Selected Persistent Symptoms in Men with Post-Covid-19 Syndrome: A Randomized Controlled Trial. J Rehabil Med. 2022; 54:jrm00330. Epub 20221020. doi: 10.2340/jrm.v54.3972. PubMed PMID: 36121338; PubMed Central PMCID: PMC9608026.
7. Pang W, Yang F, Zhao Y, Dai E, Feng J, Huang Y, et al. Qingjin Yiqi granules for post-COVID-19 condition: A randomized clinical trial. Journal of evidence-based medicine. 2022; 15(1):30–8. Epub 2022/04/14. doi: 10.1111/jebm.12465. PubMed PMID: 35416437; PubMed Central PMCID: PMC9114922.
8. Sahin H, Naz I, Karadeniz G, Sunecli O, Polat G, Ediboglu O. Effects of a home-based pulmonary rehabilitation program with and without telecoaching on health-related outcomes in COVID-19 survivors: a randomized controlled clinical study. J Bras Pneumol. 2023; 49(1):e20220107. Epub 20230123. doi: 10.36416/1806-3756/e20220107. PubMed PMID: 36700571; PubMed Central PMCID: PMC9970366.
9. Rutkowski S, Bogacz K, Czech O, Rutkowska A, Szczegielniak J. Effectiveness of an Inpatient Virtual Reality-Based Pulmonary Rehabilitation Program among COVID-19 Patients on Symptoms of Anxiety, Depression and Quality of Life: Preliminary Results from a Randomized Controlled Trial. Int J Environ Res Public Health. 2022; 19(24). Epub 20221217. doi: 10.3390/ijerph192416980. PubMed PMID: 36554860; PubMed Central PMCID: PMC9779397.
10. Rutkowski S, Bogacz K, Rutkowska A, Szczegielniak J, Casaburi R. Inpatient post-COVID-19 rehabilitation program featuring virtual reality-Preliminary results of randomized controlled trial. Front Public Health. 2023; 11:1121554. Epub 20230206. doi: 10.3389/fpubh.2023.1121554. PubMed PMID: 36815161; PubMed Central PMCID: PMC9939639.
11. Trzmiel T, Marchewka R, Pieczynska A, Zasadzka E, Zubrycki I, Kozak D, et al. The Effect of Using a Rehabilitation Robot for Patients with Post-Coronavirus Disease (COVID-19) Fatigue Syndrome. Sensors (Basel, Switzerland). 2023; 23(19). Epub 20230927. doi: 10.3390/s23198120. PubMed PMID: 37836950; PubMed Central PMCID: PMC10575211.
12. da Silva MMC, Viana DR, Colucci MG, Gonzaga LA, Arcuri JF, Frade MCM, et al. Effects of a cardiopulmonary telerehabilitation using functional exercises in individuals after COVID-19 hospital discharge: A randomized controlled trial. J Telemed Telecare. 2023:1357633X231188394. Epub 20230809. doi: 10.1177/1357633X231188394. PubMed PMID: 37559399.
13. Vieira A, Pinto A, Garcia B, Eid RAC, Mol CG, Nawa RK. Telerehabilitation improves physical function and reduces dyspnoea in people with COVID-19 and post-COVID-19 conditions: a systematic review. J Physiother. 2022; 68(2):90–8. Epub 20220409. doi: 10.1016/j.jphys.2022.03.011. PubMed PMID: 35414491; PubMed Central PMCID: PMC8994568.
14. Ahmad AM, Nawar NM, Dabess HM, Gallab MA. Effect of diaphragm manual release versus conventional breathing exercises and prone positioning on physical functional performance in women with COVID-19: A randomized trial. J Bodyw Mov Ther. 2023; 35:311–9. Epub 20230421. doi: 10.1016/j.jbmt.2023.04.064. PubMed PMID: 37330786; PubMed Central PMCID: PMC10121151.
15. Ibrahim AA, Hussein HM, Ali MS, Kanwal R, Acar T, Shaik DH, et al. A randomized controlled trial examining the impact of low vs. moderate-intensity aerobic training in post-discharge COVID-19 older subjects. Eur Rev Med Pharmacol Sci. 2023; 27(9):4280–91. doi: 10.26355/eurrev_202305_32338. PubMed PMID: 37203854.
16. Benzo RP, Ridgeway J, Hoult JP, Novotny P, Thomas BE, Lam NM, et al. Feasibility of a Health Coaching and Home-Based Rehabilitation Intervention With Remote Monitoring for COPD. Respir Care. 2021; 66(6):960–71. Epub 20210427. doi: 10.4187/respcare.08580. PubMed PMID: 33906954; PubMed Central PMCID: PMC10027177.
17. Capin JJ, Jolley SE, Morrow M, Connors M, Hare K, MaWhinney S, et al. Safety, feasibility and initial efficacy of an app-facilitated telerehabilitation (AFTER) programme for COVID-19 survivors: a pilot randomised study. BMJ open. 2022; 12(7):e061285. Epub 20220726. doi: 10.1136/bmjopen-2022-061285. PubMed PMID: 35882451; PubMed Central PMCID: PMC9329728.
18. Del Corral T, Fabero-Garrido R, Plaza-Manzano G, Fernandez-de-Las-Penas C, Navarro-Santana M, Lopez-de-Uralde-Villanueva I. Home-based respiratory muscle training on quality of life and exercise tolerance in long-term post-COVID-19: Randomized controlled trial. Ann Phys Rehabil Med. 2023; 66(1):101709. Epub 20220930. doi: 10.1016/j.rehab.2022.101709. PubMed PMID: 36191860; PubMed Central PMCID: PMC9708524.
19. Hajibashi A, Sarrafzadeh J, Amiri A, Salehi R, Vasaghi-Gharamaleki B. Effect of progressive muscle relaxation as an add-on to pulmonary telerehabilitation in discharged patients with COVID-19: A randomised controlled trial. Complement Ther Clin Pract. 2023; 51:101730. Epub 20230126. doi: 10.1016/j.ctcp.2023.101730. PubMed PMID: 36731284; PubMed Central PMCID: PMC9877153.
20. Javaherian M, Shadmehr A, Keshtkar A, Beigmohammadi MT, Dabbaghipour N, Syed A, et al. Safety and efficacy of pulmonary physiotherapy in hospitalized patients with severe COVID-19

pneumonia (PPTCOVID study): A prospective, randomised, single-blind, controlled trial. PLoS One. 2023; 18(1):e0268428. Epub 20230131. doi: 10.1371/journal.pone.0268428. PubMed PMID: 36719885; PubMed Central PMCID: PMC9888698.
21. Longobardi I, Goessler K, de Oliveira Junior GN, Prado D, Santos JVP, Meletti MM, et al. Effects of a 16-week home-based exercise training programme on health-related quality of life, functional capacity, and persistent symptoms in survivors of severe/critical COVID-19: a randomised controlled trial. Br J Sports Med. 2023; 57(20):1295–303. Epub 20230510. doi: 10.1136/bjsports-2022-106681. PubMed PMID: 37164620.
22. McNarry MA, Berg RMG, Shelley J, Hudson J, Saynor ZL, Duckers J, et al. Inspiratory muscle training enhances recovery post-COVID-19: a randomised controlled trial. Eur Respir J. 2022; 60(4). Epub 20221006. doi: 10.1183/13993003.03101-2021. PubMed PMID: 35236727; PubMed Central PMCID: PMC8900538.
23. Moon SJE, Dabbs AD, Hergenroeder AL, Vendetti ML, Jones KB, Willey BM, et al. Considerations for assessing physical function and physical activity in clinical trials during the COVID-19 pandemic. Contemp Clin Trials. 2021; 105:106407. Epub 20210420. doi: 10.1016/j.cct.2021.106407. PubMed PMID: 33887443; PubMed Central PMCID: PMC8055496.
24. Palau P, Dominguez E, Gonzalez C, Bondia E, Albiach C, Sastre C, et al. Effect of a home-based inspiratory muscle training programme on functional capacity in postdischarged patients with long COVID: the InsCOVID trial. BMJ Open Respir Res. 2022; 9(1). doi: 10.1136/bmjresp-2022-001439. PubMed PMID: 36549786; PubMed Central PMCID: PMC9791108.
25. Sedaghati P, Derakhshan KF, Ahmadabadi S, Moghaddam SRR. Effects of corrective and breathing exercises on respiratory function of older adults with a history of COVID-19 infection: a randomized controlled trial. BMC Complement Med Ther. 2023; 23(1):199. Epub 20230616. doi: 10.1186/s12906-023-04031-7. PubMed PMID: 37328735; PubMed Central PMCID: PMC10273547.
26. Tsvetkova AV, Koneva ES, Kostenko AA, Bisheva DR, Sidyakina IV, Konev SM, et al. [The role of systemic ozone therapy in the rehabilitation of patients after COVID-19]. Vopr Kurortol Fizioter Lech Fiz Kult. 2022; 99(4. Vyp. 2):22–9. doi: 10.17116/kurort20229904222. PubMed PMID: 36083814.
27. Mahler DA, Wells CK. Evaluation of clinical methods for rating dyspnea. Chest. 1988; 93(3):580–6. Epub 1988/03/01. doi: 10.1378/chest.93.3.580. PubMed PMID: 3342669.
28. American Thoracic S, American College of Chest P. ATS/ACCP Statement on cardiopulmonary exercise testing. American journal of respiratory and critical care medicine. 2003; 167(2):211–77. doi: 10.1164/rccm.167.2.211. PubMed PMID: 12524257.
29. Laboratories ATSCoPSfCPF. ATS statement: guidelines for the six-minute walk test. American journal of respiratory and critical care medicine. 2002; 166(1):111–7. doi: 10.1164/ajrccm.166.1.at1102. PubMed PMID: 12091180.
30. Holland AE, Spruit MA, Troosters T, Puhan MA, Pepin V, Saey D, et al. An official European Respiratory Society/American Thoracic Society technical standard: field walking tests in chronic respiratory disease. Eur Respir J. 2014; 44(6):1428–46. Epub 20141030. doi: 10.1183/09031936.00150314. PubMed PMID: 25359355.
31. Singh SJ, Puhan MA, Andrianopoulos V, Hernandes NA, Mitchell KE, Hill CJ, et al. An official systematic review of the European Respiratory Society/American Thoracic Society: measurement properties of field walking tests in chronic respiratory disease. Eur Respir J. 2014; 44(6):1447–78. Epub 20141030. doi: 10.1183/09031936.00150414. PubMed PMID: 25359356.
32. Criner GJ, Cordova FC, Furukawa S, Kuzma AM, Travaline JM, Leyenson V, et al. Prospective randomized trial comparing bilateral lung volume reduction surgery to pulmonary rehabilitation in severe chronic obstructive pulmonary disease. American journal of respiratory and critical care medicine. 1999; 160(6):2018–27. doi: 10.1164/ajrccm.160.6.9902117. PubMed PMID: 10588623.
33. Holden DA, Rice TW, Stelmach K, Meeker DP. Exercise testing, 6-min walk, and stair climb in the evaluation of patients at high risk for pulmonary resection. Chest. 1992; 102(6):1774–9. doi: 10.1378/chest.102.6.1774. PubMed PMID: 1446488.
34. Cahalin L, Pappagianopoulos P, Prevost S, Wain J, Ginns L. The relationship of the 6-min walk test to maximal oxygen consumption in transplant candidates with end-stage lung disease. Chest. 1995; 108(2):452–9. doi: 10.1378/chest.108.2.452. PubMed PMID: 7634883.
35. Cipriano G, Jr., Yuri D, Bernardelli GF, Mair V, Buffolo E, Branco JN. Analysis of 6-minute walk test safety in pre-heart transplantation patients. Arq Bras Cardiol. 2009; 92(4):312–9. doi: 10.1590/s0066-782x2009000400011. PubMed PMID: 19565141.
36. de Arenaza DP, Pepper J, Lees B, Rubinstein F, Nugara F, Roughton M, et al. Preoperative 6-minute walk test adds prognostic information to Euroscore in patients undergoing aortic valve replacement. Heart. 2010; 96(2):113–7. Epub 20090628. doi: 10.1136/hrt.2008.161174. PubMed PMID: 19561363.
37. Moriello C, Mayo NE, Feldman L, Carli F. Validating the six-minute walk test as a measure of recovery after elective colon resection surgery. Arch Phys Med Rehabil. 2008; 89(6):1083–9. doi: 10.1016/j.apmr.2007.11.031. PubMed PMID: 18503803.
38. Carter R, Holiday DB, Nwasuruba C, Stocks J, Grothues C, Tiep B. 6-minute walk work for assessment of functional capacity in patients with COPD. Chest. 2003; 123(5):1408–15. doi: 10.1378/chest.123.5.1408. PubMed PMID: 12740255.
39. Ben Moussa S, Rouatbi S, Ben Saad H. Incapacity, Handicap, and Oxidative Stress Markers of Male Smokers With and Without COPD. Respir Care. 2016; 61(5):668–79. Epub 20160301. doi: 10.4187/respcare.04420. PubMed PMID: 26932384.
40. Chetta A, Aiello M, Foresi A, Marangio E, D'Ippolito R, Castagnaro A, et al. Relationship between outcome measures of six-minute walk test and baseline lung function in patients with interstitial lung disease. Sarcoidosis Vasculitis and Diffuse Lung Diseases. 2001; 18(2):170–5.
41. Poulain M, Durand F, Palomba B, Ceugniet F, Desplan J, Varray A, et al. 6-minute walk testing is more sensitive than maximal incremental cycle testing for detecting oxygen desaturation in patients with COPD. Chest. 2003; 123(5):1401–7. doi: 10.1378/chest.123.5.1401. PubMed PMID: 12740254.
42. Chatterjee AB, Rissmiller RW, Meade K, Paladenech C, Conforti J, Adair NE, et al. Reproducibility of the 6-minute walk test for ambulatory oxygen prescription. Respiration. 2010; 79(2):121–7. Epub 20090520. doi: 10.1159/000220343. PubMed PMID: 19468196.
43. Callens E, Graba S, Gillet-Juvin K, Essalhi M, Bidaud-Chevalier B, Peiffer C, et al. Measurement of dynamic hyperinflation after a 6-minute walk test in patients with COPD. Chest. 2009; 136(6):1466–72. Epub 20090706. doi: 10.1378/chest.09-0410. PubMed PMID: 19581350.
44. Miyamoto S, Nagaya N, Satoh T, Kyotani S, Sakamaki F, Fujita M, et al. Clinical correlates and prognostic significance of six-minute walk test in

patients with primary pulmonary hypertension. Comparison with cardiopulmonary exercise testing. American journal of respiratory and critical care medicine. 2000; 161(2 Pt 1):487–92. doi: 10.1164/ajrccm.161.2.9906015. PubMed PMID: 10673190.
45. Kasymjanova G, Correa JA, Kreisman H, Dajczman E, Pepe C, Dobson S, et al. Prognostic value of the six-minute walk in advanced non-small cell lung cancer. J Thorac Oncol. 2009; 4(5):602–7. doi: 10.1097/JTO.0b013e31819e77e8. PubMed PMID: 19276833.
46. Lee AL, Button BM, Ellis S, Stirling R, Wilson JW, Holland AE, et al. Clinical determinants of the 6-Minute Walk Test in bronchiectasis. Respir Med. 2009; 103(5):780–5. Epub 20081212. doi: 10.1016/j.rmed.2008.11.005. PubMed PMID: 19070473.
47. Chetta A, Pisi G, Zanini A, Foresi A, Grzincich GL, Aiello M, et al. Six-minute walking test in cystic fibrosis adults with mild to moderate lung disease: comparison to healthy subjects. Respir Med. 2001; 95(12):986–91. doi: 10.1053/rmed.2001.1194. PubMed PMID: 11778797.
48. Pepin V, Brodeur J, Lacasse Y, Milot J, Leblanc P, Whittom F, et al. Six-minute walking versus shuttle walking: responsiveness to bronchodilation in chronic obstructive pulmonary disease. Thorax. 2007; 62(4):291–8. Epub 20061110. doi: 10.1136/thx.2006.065540. PubMed PMID: 17099077; PubMed Central PMCID: PMC2092479.
49. Rejbi IB, Trabelsi Y, Chouchene A, Ben Turkia W, Ben Saad H, Zbidi A, et al. Changes in six-minute walking distance during pulmonary rehabilitation in patients with COPD and in healthy subjects. Int J Chron Obstruct Pulmon Dis. 2010; 5:209–15. Epub 20100809. doi: 10.2147/copd.s7955. PubMed PMID: 20714374; PubMed Central PMCID: PMC2921688.
50. Izumizaki M, Satake M, Takahashi H, Sugawara K, Shioya T, Homma I. Effects of inspiratory muscle thixotropy on the 6-min walk distance in COPD. Respir Med. 2008; 102(7):970–7. Epub 20080418. doi: 10.1016/j.rmed.2008.02.007. PubMed PMID: 18396028.
51. Swigris JJ, Wamboldt FS, Behr J, du Bois RM, King TE, Raghu G, et al. The 6 minute walk in idiopathic pulmonary fibrosis: longitudinal changes and minimum important difference. Thorax. 2010; 65(2):173–7. Epub 20091208. doi: 10.1136/thx.2009.113498. PubMed PMID: 19996335; PubMed Central PMCID: PMC3144486.
52. Ben Saad H, Ben Hassen I, Ghannouchi I, Latiri I, Rouatbi S, Escourrou P, et al. 6-Min walk-test data in severe obstructive-sleep-apnea-hypopnea-syndrome (OSAHS) under continuous-positive-airway-pressure (CPAP) treatment. Respir Med. 2015; 109(5):642–55. Epub 20150316. doi: 10.1016/j.rmed.2015.03.001. PubMed PMID: 25820157.
53. Ben Saad H, Babba M, Boukamcha R, Ghannouchi I, Latiri I, Mezghenni S, et al. Investigation of exclusive narghile smokers: deficiency and incapacity measured by spirometry and 6-minute walk test. Respir Care. 2014; 59(11):1696–709. Epub 20140708. doi: 10.4187/respcare.03058. PubMed PMID: 25006270.
54. Shah MR, Hasselblad V, Gheorghiade M, Adams KF, Jr., Swedberg K, Califf RM, et al. Prognostic usefulness of the six-minute walk in patients with advanced congestive heart failure secondary to ischemic or nonischemic cardiomyopathy. Am J Cardiol. 2001; 88(9):987–93. doi: 10.1016/s0002-9149(01)01975-0. PubMed PMID: 11703994.
55. Kervio G, Ville NS, Leclercq C, Daubert JC, Carre F. Intensity and daily reliability of the six-minute walk test in moderate chronic heart failure patients. Arch Phys Med Rehabil. 2004; 85(9):1513–8. doi: 10.1016/j.apmr.2003.09.035. PubMed PMID: 15375827.
56. Verrill DE, Barton C, Beasley W, Lippard M, King CN. Six-minute walk performance and quality of life comparisons in North Carolina cardiac rehabilitation programs. Heart Lung. 2003; 32(1):41–51. doi: 10.1067/mhl.2003.7. PubMed PMID: 12571547.
57. Pereira de Sousa LA, Britto RR, Ribeiro AL, Baracho SM, da Costa Val Barros V, Carvalho VT, et al. Six-minute walk test in patients with permanent cardiac pacemakers. J Cardiopulm Rehabil Prev. 2008; 28(4):253–7. doi: 10.1097/01.HCR.0000327183.51992.76. PubMed PMID: 18628656.
58. Takeuchi Y, Katsuno M, Banno H, Suzuki K, Kawashima M, Atsuta N, et al. Walking capacity evaluated by the 6-minute walk test in spinal and bulbar muscular atrophy. Muscle Nerve. 2008; 38(2):964–71. doi: 10.1002/mus.21077. PubMed PMID: 18642379.
59. Falvo MJ, Earhart GM. Six-minute walk distance in persons with Parkinson disease: a hierarchical regression model. Arch Phys Med Rehabil. 2009; 90(6):1004–8. doi: 10.1016/j.apmr.2008.12.018. PubMed PMID: 19480877.
60. King S, Wessel J, Bhambhani Y, Maikala R, Sholter D, Maksymowych W. Validity and reliability of the 6 minute walk in persons with fibromyalgia. The Journal of rheumatology. 1999; 26(10):2233–7. PubMed PMID: 10529146.
61. Maanum G, Jahnsen R, Froslie KF, Larsen KL, Keller A. Walking ability and predictors of performance on the 6-minute walk test in adults with spastic cerebral palsy. Developmental medicine and child neurology. 2010; 52(6):e126–32. Epub 20100212. doi: 10.1111/j.1469-8749.2010.03614.x. PubMed PMID: 20163429.
62. Villalba WO, Sampaio-Barros PD, Pereira MC, Cerqueira EM, Leme CA, Jr., Marques-Neto JF, et al. Six-minute walk test for the evaluation of pulmonary disease severity in scleroderma patients. Chest. 2007; 131(1):217–22. doi: 10.1378/chest.06-0630. PubMed PMID: 17218579.
63. Fitts SS, Guthrie MR. Six-minute walk by people with chronic renal failure. Assessment of effort by perceived exertion. Am J Phys Med Rehabil. 1995; 74(1):54–8. doi: 10.1097/00002060-199501000-00009. PubMed PMID: 7873114.
64. Oursler KK, Katzel LI, Smith BA, Scott WB, Russ DW, Sorkin JD. Prediction of cardiorespiratory fitness in older men infected with the human immunodeficiency virus: clinical factors and value of the six-minute walk distance. J Am Geriatr Soc. 2009; 57(11):2055–61. Epub 20090928. doi: 10.1111/j.1532-5415.2009.02495.x. PubMed PMID: 19793156; PubMed Central PMCID: PMC3049945.
65. Hulens M, Vansant G, Claessens AL, Lysens R, Muls E. Predictors of 6-minute walk test results in lean, obese and morbidly obese women. Scand J Med Sci Sports. 2003; 13(2):98–105. doi: 10.1034/j.1600-0838.2003.10273.x. PubMed PMID: 12641641.
66. Hovington CL, Nadeau S, Leroux A. Comparison of walking parameters and cardiorespiratory changes during the 6-minute walk test in healthy sexagenarians and septuagenarians. Gerontology. 2009; 55(6):694–701. Epub 20090917. doi: 10.1159/000240015. PubMed PMID: 19776538.
67. Celli BR, Cote CG, Marin JM, Casanova C, Montes de Oca M, Mendez RA, et al. The body-mass index, airflow obstruction, dyspnea, and exercise capacity index in chronic obstructive pulmonary disease. N Engl J Med. 2004; 350(10):1005–12. doi: 10.1056/NEJMoa021322. PubMed PMID: 14999112.
68. Lederer DJ, Arcasoy SM, Wilt JS, D'Ovidio F, Sonett JR, Kawut SM. Six-minute-walk distance predicts waiting list survival in idiopathic pulmonary fibrosis. American journal of respiratory

and critical care medicine. 2006; 174(6):659–64. Epub 20060615. doi: 10.1164/rccm.200604-520OC. PubMed PMID: 16778159; PubMed Central PMCID: PMC2648057.
69. Latiri I, Elbey R, Hcini K, Zaoui A, Charfeddine B, Maarouf MR, et al. Six-minute walk test in non-insulin-dependent diabetes mellitus patients living in Northwest Africa. Diabetes Metab Syndr Obes. 2012; 5:227–45. Epub 20120807. doi: 10.2147 /DMSO.S28642. PubMed PMID: 22924002; PubMed Central PMCID: PMC3422904.
70. Rouatbi S, B.Saad H, Abdelghani A. The Six Minute-Walk Test is it the Most Effective Way to Follow an Overlap Syndrome? Journal of Sleep Disorders: Treatment & Care. 2013; 02(01):1–4. doi: 10.4172/2325-9639.1000108.
71. Ben Saad H, Babba M, Boukamcha R, Latiri I, Knani J, Slama R, et al. [Submaximal exercise capacity and quality of life in exclusive water-pipe smokers]. Rev Mal Respir. 2010; 27(5):489–95. Epub 20100424. doi: 10.1016/j.rmr.2010.03.009. PubMed PMID: 20569882.
72. Ben Saad H, Hamadou R, Ben Cheikh I, Chouchene A, Rejeb N, Zbidi A, et al. Réadaptation respiratoire des malades atteints d'une bronchopneumopathie chronique obstructive: données préliminaires de l'expérience tunisienne. Journal de Réadaptation Médicale: Pratique et Formation en Médecine Physique et de Réadaptation. 2008; 28(4):138–47. doi: 10.1016/j.jmr.2008.09.001.
73. Miadi-Messaoud H, Chouchane A, Ben Saad H, Debbabi H, Ben-Jebria A, Tabka Z. Six-minute walk test improved forearm skin blood flow in Tunisian obese women. Obesity (Silver Spring). 2012; 20(9):1773–9. Epub 20110630. doi: 10.1038/oby.2011.174. PubMed PMID: 21720439.
74. Abdelghani A, Ben Saad H, Ben Hassen I, Ghannouchi I, Ghrairi H, Bougmiza I, et al. [Evaluation of the deficiency and the submaximal exercise capacity in obstructive sleep apnoea patients]. Rev Mal Respir. 2010; 27(3):266–74. Epub 2010/04/03. doi: 10.1016/j.rmr.2010.02.004. PubMed PMID: 20359621.
75. Dourado VZ, Vidotto MC, Guerra RL. Reference equations for the performance of healthy adults on field walking tests. J Bras Pneumol. 2011; 37(5):607–14. doi: 10.1590/s1806-37132011000 500007. PubMed PMID: 22042392.
76. Dourado VZ. [Reference Equations for the 6-Minute Walk Test in Healthy Individuals.]. Arq Bras Cardiol. 2011; 96(6):128–38. Epub 20110225. PubMed PMID: 21359481.
77. Lataoui S, Belghali S, Zeglaoui H, Bouajina E, Ben Saad H. [Sub-maximal aerobic capacity and quality of life of patients with rheumatoid arthritis]. Rev Mal Respir. 2017; 34(1):74–85. Epub 20160914. doi: 10.1016/j.rmr.2016 .08.001. PubMed PMID: 27639946.
78. Chen H, Shi H, Liu X, Sun T, Wu J, Liu Z. Effect of Pulmonary Rehabilitation for Patients With Post-COVID-19: A Systematic Review and Meta-Analysis. Front Med (Lausanne). 2022; 9:837420. Epub 20220221. doi: 10.3389/fmed.2022.837420. PubMed PMID: 35265644; PubMed Central PMCID: PMC8899076.
79. Ahmed I, Mustafaoglu R, Yeldan I, Yasaci Z, Erhan B. Effect of Pulmonary Rehabilitation Approaches on Dyspnea, Exercise Capacity, Fatigue, Lung Functions, and Quality of Life in Patients With COVID-19: A Systematic Review and Meta-analysis. Arch Phys Med Rehabil. 2022; 103(10):2051–62. Epub 20220729. doi: 10.1016/j.apmr .2022.06.007. PubMed PMID: 35908659; PubMed Central PMCID: PMC9334878.
80. Al-Mhanna SB, Mohamed M, Noor NM, Afolabi HA, Irekeola AA, Bello KE, et al. Effectiveness of Pulmonary Rehabilitation among COVID-19 Patients: A Systematic Review and Meta-Analysis. Healthcare (Basel). 2022; 10(11). Epub 20221026. doi: 10.3390/healthcare 10112130. PubMed PMID: 36360471; PubMed Central PMCID: PMC9691193.
81. Reinert G, Muller D, Wagner P, Martinez-Pozas O, Cuenca-Zaldivar JN, Fernandez-Carnero J, et al. Pulmonary Rehabilitation in SARS-CoV-2: A Systematic Review and Meta-Analysis of Post-Acute Patients. Diagnostics (Basel). 2022; 12(12). Epub 20221202. doi: 10.3390/diagnostics 12123032. PubMed PMID: 36553039; PubMed Central PMCID: PMC9776761.
82. Rosero ID, Barreto J, Cardona C, Ordonez-Mora LT. Physical, Functional, Psychological, and Social Effects of a Physical Activity Program in Adults and Older Adults During and/or After Hospitalization for COVID-19: A Systematic Review. Risk Manag Healthc Policy. 2022; 15:2399–412. Epub 20221222. doi: 10.2147/RMHP. S386708. PubMed PMID: 36578453; PubMed Central PMCID: PMC9791952.
83. Seid AA, Aychiluhm SB, Mohammed AA. Effectiveness and feasibility of telerehabilitation in patients with COVID-19: a systematic review and meta-analysis. BMJ open. 2022; 12(10):e063961. Epub 20221005. doi: 10.1136/bmjopen-2022-063961. PubMed PMID: 36198459; PubMed Central PMCID: PMC9534777.
84. Ghram A, Latiri I, Methnani J, Souissi A, Benzarti W, Toulgui E, et al. Effects of cardiorespiratory rehabilitation program on submaximal exercise in patients with long-COVID-19 conditions: a systematic review of randomized controlled trials and recommendations for future studies. Expert Rev Respir Med. 2023; 17(12):1095–124. Epub 20231214. doi: 10. 1080/17476348.2023 .2293226. PubMed PMID: 38063359.
85. Rodriguez-Blanco C, Gonzalez-Gerez JJ, Bernal-Utrera C, Anarte-Lazo E, Perez-Ale M, Saavedra-Hernandez M. Short-Term Effects of a Conditioning Telerehabilitation Program in Confined Patients Affected by COVID-19 in the Acute Phase. A Pilot Randomized Controlled Trial. Medicina (Kaunas, Lithuania). 2021; 57(7). Epub 20210703. doi: 10.3390/medicina 57070684. PubMed PMID: 34356965; PubMed Central PMCID: PMC8305888.
86. Fereydounnia S, Shadmehr A, Tahmasbi A, Salehi RS. The Comparison of the Effectiveness of Respiratory Physiotherapy Plus Myofascial Release Therapy Versus Respiratory Physiotherapy Alone on Cardiorespiratory Parameters in Patients With COVID-19. Int J Ther Massage Bodywork. 2022; 15(1):4–14. Epub 20220302. doi: 10.3822/ijtmb.v14i4.691. PubMed PMID: 35280243; PubMed Central PMCID: PMC8887853.
87. Gonzalez-Gerez JJ, Saavedra-Hernandez M, Anarte-Lazo E, Bernal-Utrera C, Perez-Ale M, Rodriguez-Blanco C. Short-Term Effects of a Respiratory Telerehabilitation Program in Confined COVID-19 Patients in the Acute Phase: A Pilot Study. Int J Environ Res Public Health. 2021; 18(14). Epub 20210714. doi: 10.3390 /ijerph18147511. PubMed PMID: 34299962; PubMed Central PMCID: PMC8306449.
88. Pehlivan E, Palali I, Atan SG, Turan D, Cinarka H, Cetinkaya E. The effectiveness of POST-DISCHARGE telerehabilitation practices in COVID-19 patients: Tele-COVID study-randomized controlled trial. Ann Thorac Med. 2022; 17(2):110–7. Epub 20220419. doi: 10.4103/atm.atm_543_21. PubMed PMID: 35651892; PubMed Central PMCID: PMC9150661.
89. Abodonya AM, Abdelbasset WK, Awad EA, Elalfy IE, Salem HA, Elsayed SH. Inspiratory muscle training for recovered COVID-19 patients after weaning from mechanical ventilation: A pilot control clinical study. Medicine. 2021; 100(13):e25339. Epub 2021/04/01. doi: 10.1097/MD.0000 000000025339. PubMed PMID: 33787632; PubMed Central PMCID: PMC8021337.

90. Al Chikhanie Y, Veale D, Schoeffler M, Pepin JL, Verges S, Herengt F. Effectiveness of pulmonary rehabilitation in COVID-19 respiratory failure patients post-ICU. Respir Physiol Neurobiol. 2021; 287:103639. Epub 20210212. doi: 10.1016/j.resp.2021.103639. PubMed PMID: 33588090; PubMed Central PMCID: PMC7879818.
91. Busching G, Zhang Z, Schmid JP, Sigrist T, Khatami R. Effectiveness of Pulmonary Rehabilitation in Severe and Critically Ill COVID-19 Patients: A Controlled Study. Int J Environ Res Public Health. 2021; 18(17). Epub 20210825. doi: 10.3390/ijerph 18178956. PubMed PMID: 34501549; PubMed Central PMCID: PMC8430691.
92. Hayden MC, Limbach M, Schuler M, Merkl S, Schwarzl G, Jakab K, et al. Effectiveness of a Three-Week Inpatient Pulmonary Rehabilitation Program for Patients after COVID-19: A Prospective Observational Study. Int J Environ Res Public Health. 2021; 18(17). Epub 20210826. doi: 10.3390/ijerph 18179001. PubMed PMID: 34501596; PubMed Central PMCID: PMC8430843.
93. Martin I, Braem F, Baudet L, Poncin W, Fizaine S, Aboubakar F, et al. Follow-up of functional exercise capacity in patients with COVID-19: It is improved by telerehabilitation. Respir Med. 2021; 183:106438. Epub 20210430. doi: 10.1016/j.rmed.2021.106438. PubMed PMID: 33964817; PubMed Central PMCID: PMC8084600.
94. Tang Y, Jiang J, Shen P, Li M, You H, Liu C, et al. Liuzijue is a promising exercise option for rehabilitating discharged COVID-19 patients. Medicine. 2021; 100(6):e24564. doi: 10.1097 /MD.0000000000024564. PubMed PMID: 33578552; PubMed Central PMCID: PMC7886442.
95. Rodriguez-Blanco C, Bernal-Utrera C, Anarte-Lazo E, Saavedra-Hernandez M, De-La-Barrera-Aranda E, Serrera-Figallo MA, et al. Breathing exercises versus strength exercises through telerehabilitation in coronavirus disease 2019 patients in the acute phase: A randomized controlled trial. Clinical rehabilitation. 2022; 36(4):486–97. Epub 20211116. doi: 10.1177/02692155211061221. PubMed PMID: 34783270.
96. Fugazzaro S, Contri A, Esseroukh O, Kaleci S, Croci S, Massari M, et al. Rehabilitation Interventions for Post-Acute COVID-19 Syndrome: A Systematic Review. Int J Environ Res Public Health. 2022; 19(9). Epub 20220424. doi: 10.3390/ijerph 19095185. PubMed PMID: 35564579; PubMed Central PMCID: PMC9104923.
97. Ahmed I, Inam AB, Belli S, Ahmad J, Khalil W, Jafar MM. Effectiveness of aerobic exercise training program on cardio-respiratory fitness and quality of life in patients recovered from COVID-19. Eur J Physiother. 2021:1–6.
98. Iwama AM, Andrade GN, Shima P, Tanni SE, Godoy I, Dourado VZ. The six-minute walk test and body weight-walk distance product in healthy Brazilian subjects. Braz J Med Biol Res. 2009; 42(11):1080–5. Epub 20091002. doi: 10.1590/s0100-879 x2009005000032. PubMed PMID: 19802464.
99. Medicine ACoS. Acsm's guidelines for exercise testing and prescription. 9th ed2013.
100. Wootton SL, Ng LW, McKeough ZJ, Jenkins S, Hill K, Eastwood PR, et al. Ground-based walking training improves quality of life and exercise capacity in COPD. Eur Respir J. 2014; 44(4):885–94. Epub 20140819. doi: 10.1183/09031936.00078014. PubMed PMID: 25142484.
101. Zhou J, Yu Y, Cao B, Li X, Wu M, Wen T, et al. Characteristic of Clinical Studies on Baduanjin during 2000–2019: A Comprehensive Review. Evidence-based complementary and alternative medicine: eCAM. 2020; 2020:4783915. Epub 20201016. doi: 10.1155/2020/4783915. PubMed PMID: 33149753; PubMed Central PMCID: PMC7603575.
102. Szczegielniak J, Bogacz K, Majorczyk E, Szczegielniak A, Luniewski J. Post-COVID-19 rehabilitation – a Polish pilot program. Med Pr. 2021; 72(5):611–6. Epub 20210610. doi: 10.13075/mp.5893.01122. PubMed PMID: 34140706.
103. Rutkowski S, Buekers J, Rutkowska A, Cieslik B, Szczegielniak J. Monitoring Physical Activity with a Wearable Sensor in Patients with COPD during In-Hospital Pulmonary Rehabilitation Program: A Pilot Study. Sensors (Basel, Switzerland). 2021; 21(8). Epub 20210413. doi: 10.3390/s21082742. PubMed PMID: 33924536; PubMed Central PMCID: PMC8068926.
104. Zasadzka E, Tobis S, Trzmiel T, Marchewka R, Kozak D, Roksela A, et al. Application of an EMG-Rehabilitation Robot in Patients with Post-Coronavirus Fatigue Syndrome (COVID-19)-A Feasibility Study. Int J Environ Res Public Health. 2022; 19(16). Epub 20220820. doi: 10.3390/ijerph 191610398. PubMed PMID: 36012033; PubMed Central PMCID: PMC9407702.

Articles published in the Biology of Sport are licensed under an open access Creative Commons CC BY 4.0 license.
